# Local Adaptation May Help Mitigate Feminisation of Sea Turtle Populations Globally

**DOI:** 10.1111/gcb.70458

**Published:** 2025-08-29

**Authors:** Jared J. Tromp, Melissa N. Staines, Jacques‐Olivier Laloë, Graeme C. Hays

**Affiliations:** ^1^ Deakin Marine Research and Innovation Centre, School of Life and Environmental Sciences, Deakin University Geelong Victoria Australia

**Keywords:** ectotherm, endangered species, evolution, global review, global warming, marine turtle, nest site selection, operational sex ratios, resilience

## Abstract

Climate warming currently threatens many species with extinction, particularly those with a limited capacity for adaptation. Sea turtles have temperature‐dependent sex determination, whereby female hatchlings are produced at warmer incubation temperatures; hence, climate warming might cause the feminisation of populations. Recent evidence suggests that climate warming will outpace the ability of turtles to adapt through phenological shifts in nesting. Here, we examine 138 published estimates for hatchling sex ratios spanning the seven sea turtle species and all ocean basins. We evaluate whether turtles have the capacity to adapt to warming temperatures through local adaptations of the pivotal temperature at which they produce a balanced amount of male and female hatchlings. We show that at warmer sites, lower proportions of female hatchlings are produced than expected from generalised sex ratio versus incubation temperature relationships that have been previously used across all sea turtle species. This points to local adaptation of the pivotal temperature (i.e., the temperature at which a balanced hatchling sex ratio is produced) as evidenced by an analysis of 33 pivotal temperatures recorded at sites around the world that showed generally higher pivotal temperatures at warmer sites, confirming previous work. These findings point to local adaptation of the pivotal temperatures, which could help the production of male hatchlings at warmer sites and so assist with population viability. These results suggest that the sea turtle hatchling sex ratio is more resilient to climate change than previously thought.

## Graphical abstract

We evaluate whether sea turtles have the capacity to adapt to warming temperatures through local adaptations of the pivotal temperature at which they produce a balanced amount of male and female hatchlings. We show that at warmer sites, lower proportions of female hatchlings are produced than expected. These findings point to local adaptation of the pivotal temperatures, which could help the production of male hatchlings at warmer sites and so assist with population viability. These results suggest that the sea turtle hatchling sex ratio is more resilient to climate change than previously thought. Sea turtle images by NOAA Fisheries.

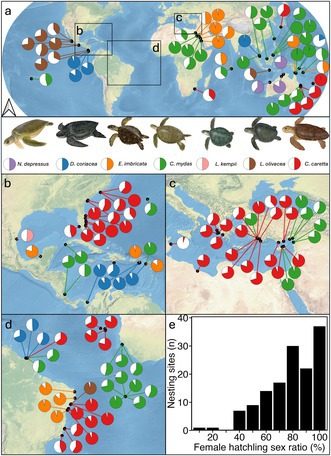

## 1 Introduction

Climate change poses significant challenges for the survival of some species and ecosystems (Malhi et al. 2020), including general warming as well as more extreme weather conditions such as heatwaves (Cheung and Frölicher 2020; Marx et al. 2021; Smale et al. 2019), droughts (Yuan et al. 2023), cyclones (Knutson et al. 2020), flooding (Wasko et al. 2021) and sea level rise (Fish et al. 2005; Galbraith et al. 2002; Hunter et al. 2015). Species that can adapt to these rapid changes are more likely to survive, for example, through changes in their geographic range, phenology (i.e., changes to the time of breeding) or physiology (Poloczanska et al. 2016). Local adaptations can occur and select for individuals in a population that produce fitter offspring, such as those with higher thermal tolerances (Howells et al. 2012).

However, for some species, there is concern that inherent components of their biology mean they are more susceptible to the adverse effects of climate warming. Sea turtles are an iconic group that have been the focus of conservation efforts across the globe (Mazaris et al. 2017). Sea turtles have temperature‐dependent sex determination, with warmer temperatures producing more females. As incubation temperatures rise due to climate change, an increased production of female hatchlings is expected (Laloë et al. 2017; Jensen et al. 2018). Ultimately, extreme feminisation and a lack of males might lead to population extinctions (Hays et al. 2017). While sea turtles may occasionally nest on new beaches and so colonise new areas (Carreras et al. 2018), their general very strong fidelity to nest on their natal beaches, that is, the area where they hatched themselves (Bowen et al. 1992; Lohmann and Lohmann 2019), means they are unlikely to shift their nesting areas quickly enough to adjust to climate warming (Hays et al. 2025). While phenological changes in the time of sea turtle nesting are known to occur (Mazaris et al. 2013; Weishampel et al. 2004), these nesting season shifts are likely insufficient to fully mitigate expected future warming (Fuentes et al. 2024; Laloë and Hays 2023). Sea turtle populations may also increase their thermal tolerance to high nest temperatures through individual variation, providing these temperatures remain below lethal limits (Kynoch et al. 2024; Pilcher et al., 2025). However, local adaptation to climate conditions might help mitigate the impacts of rapid warming. For example, if the pivotal incubation temperature, that is, the incubation temperature at which a balanced sex ratio is produced, were adaptive, then a balanced sex ratio of male and female hatchlings could be produced at different incubation temperatures. Under this scenario, sea turtles nesting in warmer climates might have a higher pivotal temperature resulting in the production of more males at warmer temperatures than sea turtles nesting in cooler climates. Recently, populations with higher pivotal temperatures have been linked to higher nest temperatures during the thermosensitive period in six of the seven extant species of sea turtles, suggesting a mechanism for thermal adaptation to local conditions in species with temperature‐dependent sex determination (Santidrián Tomillo 2024). Here, we provide a comprehensive assessment of whether pivotal temperatures are adapted to local thermal conditions using datasets collected globally, across regions and species. This analysis explores whether local adaptations could help mitigate the feminisation of hatchling production in a warming world.

## 2 Methods

### 2.1 Sea Turtle Hatchling Sex Ratios

We assembled a global database of hatchling sex ratios. We searched Google Scholar and Web of Knowledge for papers that included the terms ‘sea turtle’ and ‘sex ratio’. From the initial papers identified, we then performed forward and backward citation searches to locate additional papers. The hatchling sex ratios were summarised for each publication. Where the sex ratio was summarised by the authors, this value was used in the database, however, often the summarised sex ratios were derived from the average sex ratio across the study years or constructed from the reported table. A single hatchling sex ratio was derived, combining years, to enable global comparisons. For some publications, there was a large distance between study sites, such as in Dei Marcovaldi et al. (2016) which was based off the east coast of Brazil and spanned four states (Bahia, Sergipe, Espírito Santo and Rio de Janeiro). For these sites, a sex ratio was calculated for each state. Hatchling sex ratios were estimated using either direct or indirect measurements. Direct methods involved histological examination of the gonads or less commonly through laparoscopy, which is the physical examination of the gonads and performed on juveniles that had been collected as hatchlings and grown in captivity (e.g., Lolavar and Wyneken 2015; Wyneken et al. 2007; Wyneken and Lolavar 2015). Indirect measurements of hatchling sex ratios were derived from incubation duration, nest temperature during the second third of incubation and with a small subset inferred from mixed stock analysis (Jensen et al. 2018; Meylan et al. 2024). In order to understand the current state of natural nest hatchling sex ratios, we excluded studies that reported sex ratios that had been artificially manipulated and no longer simulated natural conditions, such as lab‐based trials with constant temperatures. Where more than one measure was reported between the direct and indirect categories in the same study (*n* = 17), the mean of the two measures was summarised to give one measurement, hereafter referred to as ‘published primary sex ratio’.

### 2.2 ICOADS Air Temperature

Air temperature records were obtained from the International Comprehensible Ocean–Atmosphere Data Set (ICOADS) through the National Center for Atmospheric Research (https://rda.ucar.edu/datasets/ds548.0/). ICOADS offers surface marine data from 1662 through to present observations that are gridded in monthly summary products in 2° latitude × 2° longitude boxes back to 1800 or 1° × 1° boxes since 1960. Data collection methods range from early noninstrumental ship observations through to modern automatic measurements from moored buoys and surface drifters. We used the Enhanced ICOADS Monthly Summary Statistics Release 3.0.0 to obtain air temperatures between January 1980 through to December 2023. Site locations were derived from latitude and longitude values reported in the study, such as site descriptions (e.g., ‘Pasture Bay, Antigua’; Mrosovsky et al. 1992) or study maps (e.g., Bently et al., 2020). From the 1° × 1° ICOADS pixels, we summarised a 2° × 2° contiguous area that was centred around each GPS coordinate as a close proxy of the adjoining beach environment. Although there are differences between beaches and species (e.g., due to nest depths), on average a 1°C increase in air temperature equates to a 0.86°C in sand temperature at nest depths (Laloë, Chivers, et al. 2021) and so historical and predicted changes in air temperature likely provide a good indication of past and future changes in sand temperatures at nests depths (Esteban et al. 2016; Hays et al. 2003). Monthly means that had less than 10 observations were excluded from the calculations, following previous data cleaning methods (Fuentes et al. 2009; Hays et al. 2003). In some instances, we were unable to obtain values in all four 1° × 1° pixels. For these sites (i.e., Godfrey and Mrosovsky 2006; Leh et al. 1985; Mrosovsky et al. 1984) the closest adjacent 2° × 2° contiguous pixel was used as a proxy. The top 25% of the warmest temperatures within the 2° × 2° contiguous pixel were used to approximate nesting temperatures given that sea turtles typically nest during the warmer summer months. From the top 25% of warmest air temperatures, a weighted mean was calculated by the following formula:

$${\bar{x}}_{\text{weighted}}=\frac{\sum_{i=1}^{k}\left({\bar{x}}_{i}\cdot n_{i}\right)\;}{\sum_{i=1}^{k}n_{i}}$$

where the monthly mean temperature (${\bar{x}}_{i}$) in each 1° × 1° pixel is multiplied by the number of monthly observations ($n_{i}$). This gives the weighted mean across the 2° × 2° contiguous pixel for the top 25% of warmest monthly ICOADS temperatures, hereafter referred to as ‘monthly air temperature’. Where there were less than 3 months of observed temperature values from the four 1° × 1° ICOADS pixels within the study year(s), that is, the year(s) sampling occurred, we expanded the number of years before and after the study until we could achieve a minimum of three‐monthly means. In addition, we added an additional year before and after the study breeding year(s), for breeding seasons that overlapped 2 years, such as those in the Southern hemisphere where the summer months are in December, January and February. Where turtles nested outside of the summer months, we restricted analysis to only include monthly air temperatures recorded within the breeding season months, accounting for biologically relevant nesting temperatures. We compiled a database of breeding seasons that were derived from descriptors in the source study, inferred from existing compiled sea turtle nesting seasons in Laloë et al. (2023) or from the regional proximity of the same species. Finally, where there were insufficient data for turtles nesting outside of the summer months, such as Maulany et al. (2012), we used all available data points within the 2° × 2° contiguous pixel (*n* = 18).

### 2.3 Predicting Hatchling Sex Ratios

Using the published relationships (*n* = 36) between air and sand temperature at nesting depth (compiled in Laloë, Chivers, et al. 2021), we predicted the sand temperatures from ICOADS air temperatures at each site. Sex ratio study sites were matched to sand/air temperature beaches if they were within 450 km. Where two or more linear relationships were listed for the same site, that is, Ascension Island, the relationships were randomly selected, maintaining an even representation of air and sand temperature relationships between sex ratio study sites. We predicted hatchling sex ratios from air and sand temperature relationships in three ways: (a) First, all sand versus air temperature relationships were randomly assigned across all sites, representing the largest amount of residual error in the model. (b) Second, we used site‐specific sand versus air temperature relationships where they existed and randomly selected a sand versus air temperature relationship where site‐specific relationship did not exist. (c) Thirdly, we again used site‐specific relationships where available and then the global mean sand versus air temperature relationship reported by Laloë, Chivers, et al. (2021) where a site‐specific relationship was not available. For each predicted sand temperature, we added 1.1°C to account for metabolic heating within the nest (Hays et al. 2021). With the derived sand temperature, we used a generalised logistic model (Hays et al. 2017) to predict hatchling sex ratios, hereafter referred to as ‘predicted sex ratios’.

### 2.4 Pivotal Temperature

We performed a literature review on the pivotal temperature of hatchling sex ratios in sea turtles using search terms of ‘pivotal temperature’, ‘sea turtle’, ‘hatchling sex ratio’ and completed forward and reverse citation searches of key literature and published pivotal temperature tables. Forty‐six pivotal temperatures were collated, 13 derived their temperatures from field studies and 33 were experimentally derived using constant temperature incubation chambers. Field studies were excluded from the analysis. Pivotal temperatures were estimated to give a single value to enable formal analysis: for example, when a mixed ratio was reported between 28.5°C and 30.3°C, we calculated the mean of the range giving a pivotal temperature of 29.4°C (Standora and Spotila 1985); when a study ‘suggest[s] a pivotal temperature near 31 [°]C’ (Wibbels et al. 1998), we used that value as the pivotal temperature estimate. Air temperature was derived as outlined above, selecting the top 25% of warmest values from 1980 to 2023 within a 2° × 2° contiguous pixel; with a linear model used to test the relationship between pivotal temperature and ICOADS weighted mean air temperature. Visual assessment of residual patterns was used to check assumptions of statistical tests. Data were analysed in R v4.4.2 using RStudio v2024.04.2 (R Core Team, 2023) and maps were produced using QGIS v3.36.2.

## 3 Results

### 3.1 Empirical Data on Sea Turtle Hatchling Sex Ratios

**FIGURE 1:**
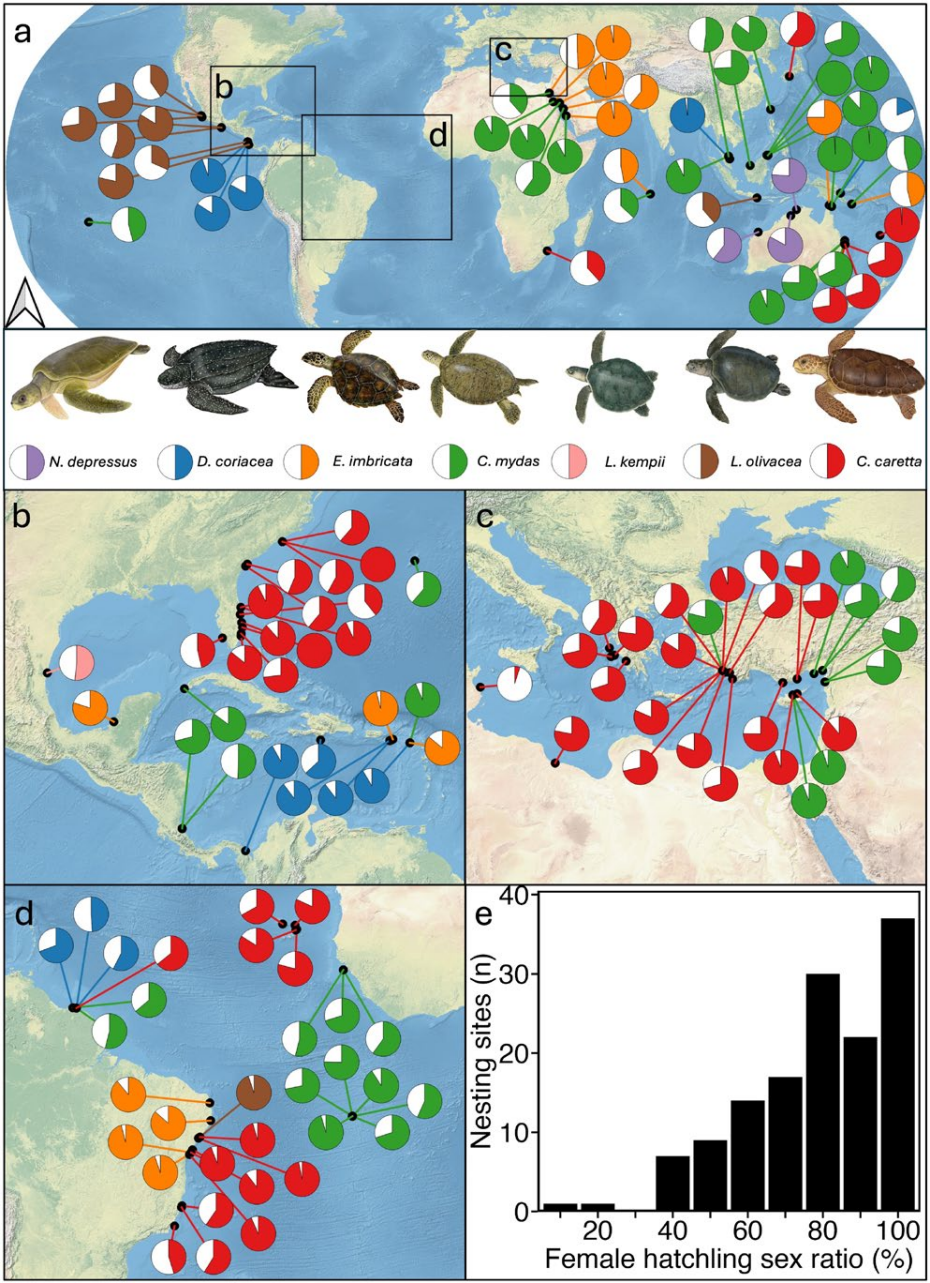
Primary sex ratios of the seven extant sea turtles recorded (*n* = 138) from (a) global, (b) Caribbean, (c) Mediterranean and (d) Atlantic populations. Turtle species are identified through coloured pie charts with the solid colour representing the percentage female hatchling sex ratio reported in the literature. Black points indicate the location of where the study took place and can represent multiple research papers, years and beaches. (e) The distribution of female hatchling sex ratios is presented in 10% bins with the highest number of nesting sites showing a primary sex ratio < 90%. Sea turtle images by NOAA Fisheries.

We compiled 138 primary hatchling sex ratios for sea turtle populations from 108 published studies, including data from all seven species and from the Atlantic, Pacific and Indian Oceans as well as the Mediterranean and Caribbean (Figure 1a–d). Overall hatchling sex ratios tended to be female biased (Figure 1), whereby 106 of 138 records are 60% female or higher, with only 9 of 138 records less than 40% (Figure 1e). Strong female biased populations are seen in 59 of 138 sites, where hatchling sex ratios were ≥ 80% female, and at 37 of 138 sites, where they are ≥ 90% female. At some sites, massively skewed female hatchling sex ratios (> 90% female) have been reported. For example, a green turtle site in the northern Great Barrier Reef of Australia (~99% female) (Jensen et al. 2018), as well as a loggerhead ( *Caretta caretta* ) hawksbill ( *Eretmochelys imbricata* ) and olive ridley turtle ( *Lepidochelys olivacea* ) site off the east coast of Brazil (93%–96% female) (Castheloge et al. 2018; Dei Marcovaldi et al. 2014, 2016) and a loggerhead turtle site in Florida, USA (93% female). There were 51 of 138 sites where the hatchling sex ratio was primarily balanced between 30% and 70% female. There tended to be fewer sites where hatchling sex ratios were strongly male biased. For example, < 20% female hatchling sex ratios were only reported in 2 of 138 sites. These include loggerhead turtles in the Kuriat islands of Tunisia (5% female) (Jribi and Bradai 2014) and leatherback turtles ( *Dermochelys coriacea* ) off the Huon coast of Papua New Guinea (19% female) (Steckenreuter et al. 2010). Generally, female biased hatchling sex ratios dominated across regions. For example, the proportion of reports of > 80% female hatchling was 14 of 32 for the Atlantic (Figure 1d), 10 of 28 for the Mediterranean (Figure 1c), 14 of 27 for Caribbean (Figure 1b) and 21 of 51 from the remaining locations (Figure 1a). Green and loggerhead turtles comprised over 70% of the sex ratios, with few studies reporting on olive ridley (*n* = 9), Kemp's ridley ( *Lepidochelys kempii* ) (*n* = 1) and flatback turtles ( *Natator depressus* ) (*n* = 3). A similar female bias is apparent between the species, where the proportion of reports with skewed female hatchling sex ratios (> 80% female) for loggerhead turtles was 38.5% (20 of 52), 42.2% for green turtles (19 of 45), 61.5% for leatherback turtles (8 of 13), 60% for hawksbill turtles (9 of 15), 22.2% for olive ridley turtles (2 of 9), 33.3% for flatback turtles (1 of 3) and one study on Kemp's ridley turtles reporting a balanced sex ratio (51.65% female) (Bevan 2013). There is a moderate sampling bias in the Mediterranean, Atlantic and Caribbean, which represent 87 of the 138 sex ratio records (63%). The database spans several decades, with research published from 1982 through to the present.

### 3.2 Reliability of Hatchling Sex Ratio Estimates

When compiling the hatchling sex ratio database, we generalised the sex ratio values into a single summary that could be used to represent the study site population for formal analysis. Due to the variety of methods, generalised thermal reaction norms, years, sites and sample sizes used to measure or estimate sex ratios across a population, there is inherent variability in the database; yet, the biased or balanced sex ratios are apparent across all measures. For example, at one site in Turkey (Anamur), the calculated mean sex ratio from the temperature during middle third incubation was 64.6% female and from the incubation duration to be 85.2% female, with the histological sex ratio of dead and late‐stage embryos averaging 75.6% (2006: 72.1% (*n* = 366); 2007: 79% (*n* = 271)) female (Uçar et al. 2012). Similarly, Candan and Kolankaya (2016) showed high agreement between direct sex ratio measurements of 56.4% (*n* = 188) and indirect estimates which ranged from 57.4% (*n* = 12) to 58.5% (*n* = 12) female. Even with the most extreme differences in reported hatchling sex ratios using direct and indirect measures, there was still a broad consensus in the conclusions. For example, Schmid et al. (2008) reported the largest difference between the direct (57.6% female) and indirect (34.9% female) sex ratio estimates. Taken together, these values are indicative of a balanced hatchling sex ratio depending on the year. The percentage difference within indirect measures of sex ratios (estimates recorded in the same study) ranged from 1.9% to 27.5% (*n* = 4). Between direct and indirect measurements, there was a discordance of 0.9%–48.9% (*n* = 16). So, while identifying the true hatchling sex ratios from beaches is challenging, generally, conclusions of balanced versus extreme male or female biases are likely robust.

### 3.3 Residual Patterns in Hatchling Sex Ratios Predicted From Air Temperature

**FIGURE 2:**
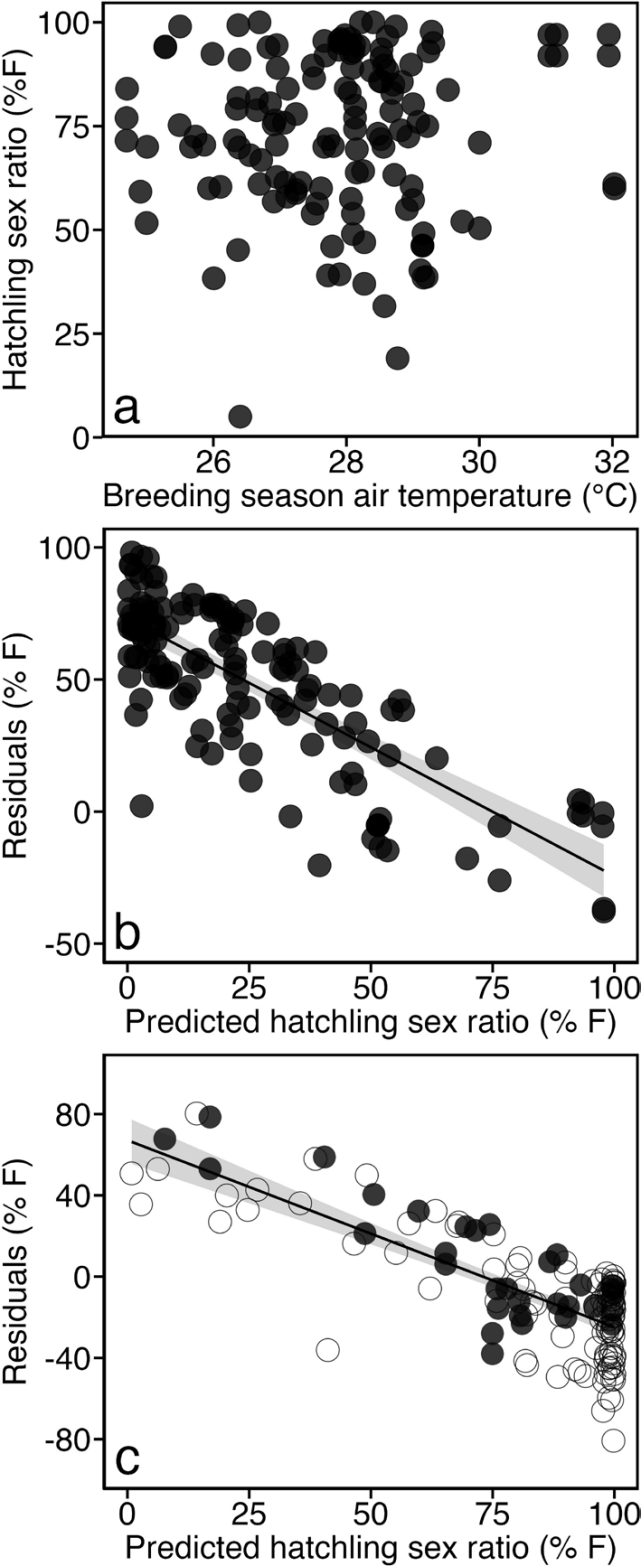
(a) Global hatchling sex ratios derived from the literature plotted against breeding season ICOADS air temperature, we found no linear relationship between air temperature and hatchling sex ratio (*R* ^2^ < 0.01; *F*_1,136_ = 0.2747, *p* = 0.6011). (b) Hatchling sex ratios were predicted based on the logistic relationship (Hays et al. 2017) between ICOADS air temperature and global pivotal temperature (29.1°C), showing a strong linear relationship (*R* ^2^ = 0.6172; *F*_1,136_ = 219.3, *p* < 0.001) between the residuals (published primary vs. predicted hatchling sex ratio) and hatchling sex ratio, solid line represents the linear model with 95% confidence intervals represented in grey shading. (c) Predicted global hatchling sex ratios based on known air and sand temperature relationships (Laloë, Chivers, et al. 2021) with metabolic heating (Hays et al. 2021) showed a strong linear relationship (*R* ^2^ = 0.5489; *F*_1,136_ = 165.5, *p* < 0.001) between the residuals (published primary vs. predicted hatchling sex ratio) from the predicted sex ratio. Closed circles indicate that the study site was matched to a beach with a known air and sand temperature relationship, while open circles were based on a randomly selected relationship.

When we compared the reported hatchling sex ratio for each site against monthly air temperature, we found no relationship (*R* ^2^ < 0.01; F_1,136_ = 0.2747, *p* = 0.601). Monthly air temperatures > 30°C were matched to a range of hatchling sex ratios from 50.4% to 97% female (Figure 2a). Similarly, at lower monthly air temperatures < 27°C, there was a wide range in hatchling sex ratios from 5% to 100% female. Monthly air temperatures ranged from 24.7°C to 32°C across the study sites. Considering the lack of a clear relationship between hatchling sex ratio and monthly air temperature, we explored the residual variation or the difference between predicted and published primary hatchling sex ratios when we either (a) assumed sand temperature equalled air temperature (*R* ^2^ = 0.6172; F_1,136_ = 219.3, *p* < 0.001) or (b) converted air temperature to sand temperature and added metabolic heating using the three procedures outlined in the methods that used known sand versus air temperature relationships. Using either approach, we found significant relationships (*p* < 0.001, *R* ^2^ ranged between 0.467 and 0.6653) between the residuals of predicted sex ratio (Figure 2b,c) and temperature, where more females were observed at lower temperatures than predicted (positive residual) and fewer females were observed at higher temperatures than predicted (negative residual).

### 3.4 Local Adaptation of Pivotal Temperature

**FIGURE 3:**
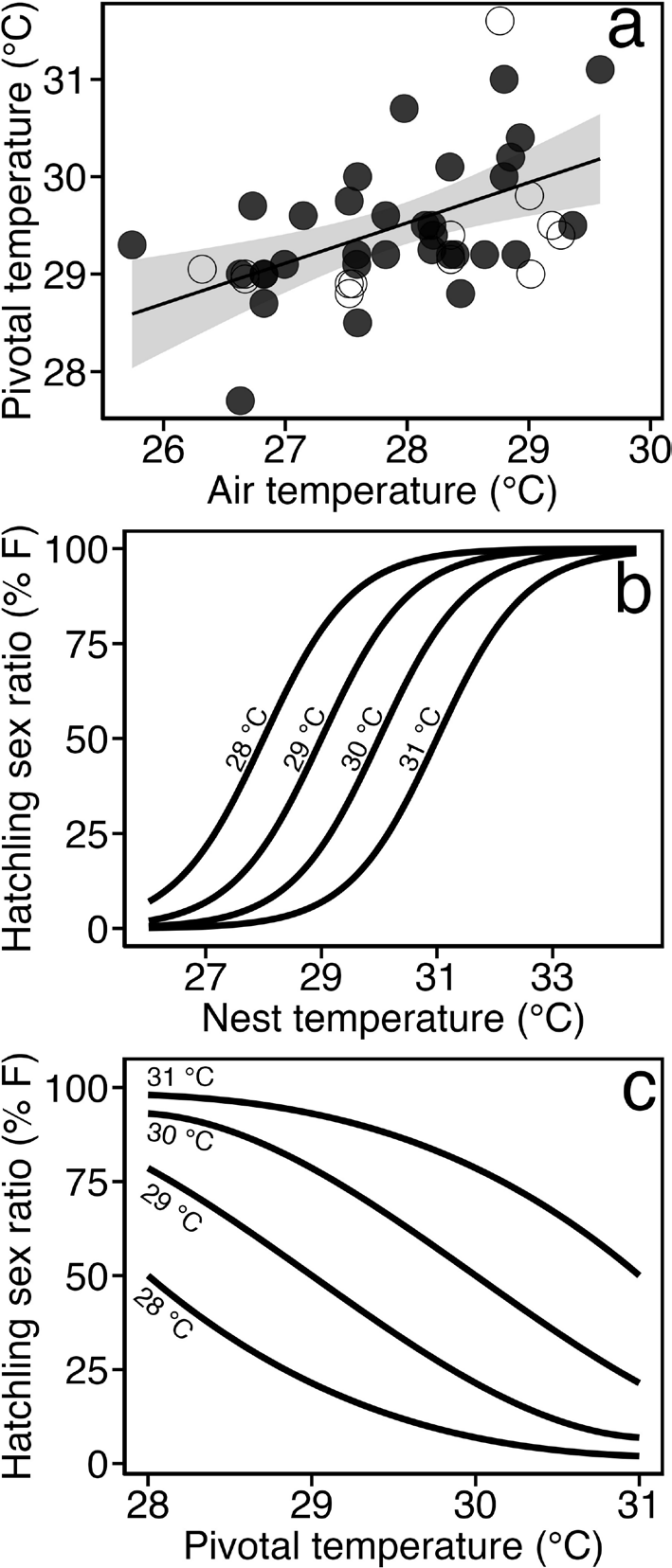
(a) Global sea turtle pivotal temperatures derived from the literature plotted against monthly air temperature. A linear model (solid black line) was fitted with 95% confidence intervals (grey intervals) and showed a significant positive relationship (*R* ^2^ = 0.2861; *F*_1,31_ = 12.42, *p* < 0.001) between air temperature and pivotal temperature. Filled circles represent studies in which pivotal temperatures were derived from controlled laboratory studies and open circles represent pivotal temperatures inferred from field‐based studies. Field studies are plotted but not included in the linear regression. (b) Modelled logistic hatchling sex ratio curves over a range of nest temperatures considering four pivotal temperatures (28°C, 29°C, 30°C and 31°C). (c) Thermosensitive period profiles of hatchling sex ratios plotted against four pivotal temperatures (28°C, 29°C, 30°C and 31°C), estimating the sex ratio from the nest temperature across the pivotal temperatures.

We compiled 33 laboratory‐derived and 13 field‐based pivotal temperatures to consider the influence of local conditions on pivotal temperature. Using the location data from the studies, we were able to match these with monthly air temperatures to represent the temperature within a study region. We found a significant linear trend (*R* ^2^ < 0.2861; *F*_1,31_ = 12.42, *p* < 0.001) between experimentally derived pivotal temperatures and monthly air temperature, indicating that pivotal temperature increases at sites with hotter air temperatures (Figure 3a). We report a range of pivotal temperatures from 27.7°C–31.1°C with a difference of up to 3.4°C between species and up to 2.3°C within species, with higher pivotal temperatures found at sites experiencing higher air temperatures. Considering the variation in pivotal temperatures, we modelled the logistic hatchling sex ratios curves over a range of nest temperatures, considering four (i.e., at 28°C, 29°C, 30°C and 31°C) pivotal temperatures (Figure 3b). Given these logistic curves, we predicted the range for which local adaptation could modulate hatchling sex ratios based on the temperature during the thermosensitive period across differing population‐specific pivotal temperatures (Figure 3c). As such, changes in population‐specific pivotal temperatures can have profound impacts on estimated sex ratios, that is, comparing pivotal temperatures of 28°C, 29°C, 30°C and 31°C at a nest temperature of 29°C (simulating the thermosensitive period) would produce female hatchling sex ratios of 78.6%, 50%, 21.4% and 6.9% respectively. Considering this variation, there is a window whereby populations could adapt to increased nest temperatures by increasing their pivotal temperature through local adaptation, thereby producing fewer females and having a meaningful impact on population‐level hatchling sex ratios. However, extreme nest temperatures below 26°C or above 33°C during the thermosensitive period provide little opportunity to buffer the impact of climate change in the hatchling sex ratio (< 7% and > 93% female respectively) regardless of the pivotal temperature used.

## 4 Discussion

Climate change impacts have been a concern for species with temperature‐dependent sex determination for over half a century (Bock et al. 2020; Honeycutt et al. 2019; Mrosovsky 1994; Valenzuela et al. 2019). For sea turtles, the effect of warming incubation temperatures on sex ratios has been a growing field of research in recent years (Hays et al. 2022; Hays et al. 2023; Fuentes et al. 2023; Fuentes et al. 2024), especially given that female‐biased sex ratios are being increasingly reported at nesting sites across the world (Hays et al. 2017; Jensen et al. 2018; Laloë et al. 2016). Previous research has shown that behavioural adaptations such as shifts in the nesting season to cooler months and colonisation of new nesting sites can mitigate warming temperatures to an extent (Booth et al. 2020; Fuentes et al. 2024; Laloë and Hays 2023; Santidrián Tomillo et al. 2023; Tello‐Sahagún et al. 2023). Here, we show that alongside behavioural adaptations, sea turtles may be able to adapt to warming temperatures through evolutionary adaptations, namely, through a shift of the pivotal temperature for temperature‐dependent sex determination. This work confirms previously demonstrated relationships of higher pivotal temperatures found in populations with warmer nest temperatures and, alongside other evolutionary adaptations such as increased thermal tolerance, may ameliorate some of the impacts of climate change (Kynoch et al. 2024; Santidrián Tomillo 2024). However, at some nesting sites around the globe, that is, Raine Island (99% female hatchling sex ratio), the rate of change in temperature may already be beyond the capacity of turtles to adapt (Laloë and Hays 2023).

Our global hatchling sex ratio database includes values from all seven sea turtles, although the species are not equally represented. Loggerhead and green turtles make up the majority of entries, with flatback, olive ridley and Kemp's ridley turtle being underrepresented, likely reflecting their conservation status and endemicity. The Mediterranean, Caribbean and Atlantic provide well over half of the published sex ratios, with few studies reporting sex ratios in the Indian Ocean, despite this being a major nesting site for hawksbill and green turtles. Our findings reiterate and extend previous compilations showing hatchling sex ratios of sea turtles around the globe and across species are generally female‐biased (Hays et al. 2017; Hays et al. 2014; Laloë et al. 2023; Figure 1). This is of concern, as the rate of temperature increase, through climate change, is likely to outpace the ability of turtles and other taxa with temperature‐dependent sex determination (i.e., reptiles and fish) to adapt. While initially, increased proportions of female hatchlings are likely to bolster population growth, providing more opportunities for males to mate (Hays et al. 2017; Santidrián Tomillo 2022; Hays et al., 2022), and providing opportunity for population growth through an increase in nest numbers and egg production (Heppell et al. 2022; Wedekind 2002). Long‐term female‐biased production could significantly challenge the availability of males during the breeding season (Hays et al. 2017). However, female‐biased primary sex ratios do not necessarily translate into a female‐skewed operational (breeding) sex ratio (Staines et al. 2022), as males tend to return to breed two to three times more frequently than females (Hays et al. 2010, 2014; Wright et al. 2012). Projected climate warming scenarios have demonstrated that sea turtle populations are becoming increasingly female‐biased, even under moderate CO_2_ emission scenarios (Blechschmidt et al. 2020). Hence, there is an urgent need to assess methods by which sea turtles might mitigate feminisation of populations or if intervention is needed to cool nests (Fuentes et al. 2024).

Despite the global threat of feminised populations, our analysis shows that there are still sites across all major regions that produce balanced sex ratios and uniquely two that demonstrate a strong male bias (Jribi and Bradai 2014; Steckenreuter et al. 2010). These include loggerhead turtles on the northern coast of Africa in the Mediterranean and leatherbacks on the east coast of Papua New Guinea. Recently, white sand islands located in the Asia‐Pacific region have been highlighted as potential climate change refugia, given evidence of relatively balanced and slight female bias estimated for primary sex ratios from environmental proxies (Staines et al. 2023; Laloe et al. 2020). These sites demonstrate the capacity for unbiased sex ratio production and highlight the need to investigate hatchling sex ratios in underrepresented regions and species.

While there are very well‐established relationships between air and sand temperature at individual nesting beaches (Bentley et al. 2020; Fuentes et al. 2009, 2010; Laloë et al. 2014; Özdemir et al. 2011), our results suggest that there is no direct relationship between mean monthly air temperature and primary hatchling sex ratio when comparing sites across the globe. There are likely several reasons for this lack of relationship globally. For example, in addition to air temperature, other factors such as the colour of sand (albedo), the extent of seasonal rainfall, shading and beach orientation can all influence sand temperature (Booth and Freeman 2006; Hays et al. 2001; Houghton et al. 2007; Reboul et al. 2021; Staines et al. 2019, 2020) and differences in these factors between sites might override the role of air temperature. There are also biological factors, as it has been previously demonstrated that deeper nests are relatively cooler than shallower nests and experience less daily temperature variation (Booth and Freeman 2006; van de Merwe et al. 2005). Metabolic heating also varies between sites, species and likely between clutches (Gammon et al., 2020; Zbinden et al. 2006). These other factors might be expected to simply introduce noise in the relationship between air temperature and hatchling sex ratio. While a study on the green turtles ( *Chelonia mydas* ) nesting on Ascension Island suggested local adaptation to darker, warmer beaches versus light coloured, cooler beaches (Weber et al. 2012), a follow‐up study by the same research group showed no evidence of any local adaptation (Tilley et al. 2019). Further, the initial results were unexpected given the proximity of these warmer versus cooler beaches (a few kilometres apart); therefore, their likely lack of genetic separation to allow the evolution of local adaptation is noted. However, for nesting beaches that are more distantly separated and hence where individuals using different nesting beaches are genetically isolated due to natal philopatry, then it is more conceivable that there might be local adaptation, considering the direct link between pivotal temperature and nest temperatures for several sea turtle populations and species around the world (Santidrián Tomillo 2024). Our finding that at warm sites fewer than expected female hatchings were observed and vice versa suggests that there may be local adaptation of hatchling sex ratios. One possible explanation for this finding is local adaptation of the pivotal temperatures, an explanation supported by our examination of 33 pivotal temperatures reported at sites around the world, where we found higher pivotal temperatures at warmer sites. Taken together, our results and those of Santidrián Tomillo (2024) suggest that the pivotal temperature is plastic and that turtles nesting in warmer sites have evolved to have a higher pivotal temperature as an adaptation to their local environment.

The ability for sea turtles to alter their pivotal temperature in response to climate variability could potentially have significant implications for their survival in the face of climate change. Rapid evolutionary adaptations have previously been observed in species with short generation times (e.g., plankton; O'Dea et al. 2014). However, sea turtles have long generation times (i.e., several decades; Schofield et al. 2020) so it is unknown how quickly they could adapt to new environmental conditions. Additionally, little is known about the genetic variation of the pivotal temperature within a population, as this could substantially impact a population's ability to adapt. A recent study by Porter et al. (2021) used constant temperature experiments to determine the window of time during the middle third of development when hatchling sex could be manipulated (i.e., the thermal sensitive period). In doing so, green turtle eggs were collected from four different nesting females; however, one clutch had a greater propensity to produce males compared to the others (Porter et al. 2021). Despite the known pivotal temperature for the southern Great Barrier Reef stock being 28.1°C (Booth et al. 2004), an average of 80% male hatchlings were produced from a clutch at female‐producing temperatures (29°C–29.7°C), while the average across three other clutches was only 17% male (Porter et al. 2021). From this study, it is clear that more research is needed to investigate the genetic components of temperature‐dependent sex determination and how that could play a role in a population's ability to adapt to a rapidly warming climate.

Our theoretical considerations show that there is a clear range in nest temperatures where local adaptation of pivotal temperature could provide a substantive buffer against extreme bias in hatchling sex ratios. For example, at intermediate nest temperatures, such as 29°C during the thermosensitive period, female hatchling sex ratios can range from 21.4% to 93.1%, dependent on the pivotal temperature. Local adaptation of the pivotal temperature might raise the chances that, even in populations that generally produce mostly female hatchlings, there could be occasional cooler years that produce enough males to sustain populations (Heppell et al. 2022). Indeed, nest temperatures have been shown to vary seasonally, demonstrating that male‐producing years are possible (Binckley et al. 1998). Furthermore, enough males to sustain populations may also result from more male production, in cooler conditions at the start and end of the nesting season (Lolavar and Wyneken 2015; Rebelo et al. 2012; Sarı and Kaska 2016; Tanabe et al. 2020). Finally, inherent variability within a population may result in the occasional male‐biased nest amongst a female‐biased nesting beach (Booth and Astill 2001; Kaska et al. 2006). As such, further work is needed to understand the relative role of the adaptation of the pivotal temperature as a means to mitigate feminisation of populations (Patrício et al. 2017); compared to other processes such as shifts in phenology, interannual variability in temperatures and long‐term increases in either rainfall (Houghton et al. 2007) or extreme rainfall events (Staines et al. 2020; Laloë, Tedeschi, et al. 2021).

In conclusion, here we provide an update to both the global hatchling sex ratio and pivotal temperature databases that can be explored to further understand global sea turtle hatchling sex ratios. Our results suggest that the pivotal temperature for temperature‐dependent sex determination in sea turtles is plastic and may be adaptive, with higher pivotal temperatures found at sites experiencing higher air temperatures, confirming previous findings. Hence, sea turtles might be able to mitigate warming temperatures through evolutionary adaptation. However, one unresolved question is how quickly the pivotal temperature adjusted to new conditions in the past and whether sea turtles can adapt to the current rate of climate change. Further research should investigate population‐specific pivotal temperatures and their influence on primary sex ratios.

## Author Contributions

**Jared J. Tromp:** data curation, formal analysis, investigation, methodology, project administration, visualization, writing – original draft. **Melissa N. Staines:** data curation, investigation, writing – review and editing. **Jacques‐Olivier Laloë:** data curation, investigation, writing – review and editing. **Graeme C. Hays:** conceptualization, formal analysis, investigation, methodology, writing – review and editing.

## Conflicts of Interest

The authors declare no conflicts of interest.

## Supporting information


**Table S1:** Sea turtle hatching sex ratios for key nesting sites around the globe with derived monthly air temperatures (ICOADS), representing the thermal conditions during the nesting period.

**Table S2:** Sea turtle pivotal temperatures of key nesting sites around the globe with derived monthly air temperatures (ICOADS), representing the thermal conditions during the nesting period. Pivotal temperatures were either experimentally determined in a temperature‐controlled environment (laboratory) or calculated in situ (field study).

**Table S3:** Sea turtle nesting season of key nesting sites around the globe. Months highlighted in bold indicate the peak of nesting season as given in the reference.

## Data Availability

The data that support the findings of this study are openly available in Dryad at https://doi.org/10.5061/dryad.dncjsxmbq.
