# Supplementary material for: Local Adaptation May Help Mitigate Feminisation of Sea Turtle Populations Globally
Source: Glob Chang Biol. 2025 Aug 29;31(9):e70458. doi: 10.1111/gcb.70458 (PMC12396736; doi:10.1111/gcb.70458)
Supplement: Supplementary file 1 — Table S1: Sea turtle hatching sex ratios for key nesting sites around the globe with derived monthly air temperatures (ICOADS), representing the thermal conditions during the nesting period. [file GCB-31-e70458-s002.pdf]

Title: Local adaptation may help mitigate feminisation of sea turtle populations globally

Jared J. Tromp*, Melissa N. Staines, Jacques-Olivier Laloë and Graeme C. Hays
*Corresponding author E-mail: j.tromp@deakin.edu.au

Supplementary table 1

Table S1: Sea turtle hatching sex ratios for key nesting sites around the globe with derived monthly air temperatures (ICOADS), representing the thermal conditions during the nesting period.

First Author	Year	Site	Region RMU 2023	Species	Sex ratio	Monthly air temperature
Godley	2001	Alagadi, North Cyprus	Mediterranean	Cc	94.00	25.28
Broderick	2000	Alagadi, North Cyprus	Mediterranean	Cm	94.17	25.28
Uçar	2012	Anamur, Turkey	Mediterranean	Cc	75.23	25.49
Perez	2016	Boa Vista, Cape Verde Islands	Atlantic, Northeast	Cc	79.15	26.35
Patino-Martinez	2011	Capitancito, Columbia	Atlantic, Northwest	Dc	92.00	27.69
Sari	2015	Dalyan, Turkey	Mediterranean	Cc	60.38	26.11
Ozdemir	2011	Dalyan, Turkey	Mediterranean	Cc	71.58	26.32
Ilgaz	2011	Dalyan, Turkey	Mediterranean	Cc	84.00	27.12
Kaska	1998	Akdeniz, Cyprus	Mediterranean	Cc	81.63	26.65
Kaska	1998	Akdeniz, Cyprus	Mediterranean	Cm	78.80	26.65
Blamires	2003	Fog bay, Australia	Indian, Southeast	Nd	76.00	29.08
Booth	2006	Heron Island, Australia	Pacific, Southwest	Cm	94.00	26.83
Booth	2001	Heron Island, Australia	Pacific, Southwest	Cm	75.80	27.06
Loop	1995	Milman Island, Australia	Pacific, Southwest	Ei	75.00	29.22
Wood	2014	Mon Repos, Ausltralia	Pacific, South	Cc	72.48	25.73
Chu	2008	Mon Repos, Ausltralia	Pacific, South	Cc	69.84	26.39
Jensen	2018	nGBR, Australia	Pacific, Southwest	Cm	99.00	28.74

King	2013	Lanyu Island, Taiwan	East Indian and Southeast Asia	Cm	85.67	28.83
Katselidis	2012	Zakynthos island, Greece	Mediterranean	Cc	76.93	24.69
Zbinden	2007	Zakynthos island, Greece	Mediterranean	Cc	71.50	24.69
Laloë	2016	St Eustatius, Dutch Caribbean	Atlantic, North	Cm	93.50	28.53
Laloë	2016	St Eustatius, Dutch Caribbean	Atlantic, Northwest	Ei	85.90	28.50
Laloë	2016	St Eustatius, Dutch Caribbean	Atlantic, Northwest	Dc	91.50	28.53
Marcovaldi	1997	Sergipe, Brazil	Atlantic, Southwest	Cc	96.90	27.98
Marcovaldi	1997	Bahia, Brazil	Atlantic, Southwest	Cc	94.27	28.19
Marcovaldi	1997	Espirito Santo, Brazil	Atlantic, Southwest	Cc	59.63	27.24
Godfrey	1999	Bahia, Brazil	Atlantic, Southwest	Ei	94.78	28.02
Marcovaldi	2014	Bahia, Brazil	Atlantic, Southwest	Ei	96.00	27.92
Marcovaldi	2014	Rio Grade do Norte, Brazil	Atlantic, Southwest	Ei	89.00	28.09
Maulany	2012	Alas Purwo National Park, Indonesia	Pacific, West	Lo	38.70	29.21
Mrosovsky	1989	Cape Canaveral Air Force Station, Florida	Atlantic, Northwest	Cc	93.00	28.11
Mrosovsky	1992	Cape Canaveral Air Force Station, Florida	Atlantic, Northwest	Cc	92.55	28.07
Broderick	2001	Ascension Island, British Overseas Territory	Atlantic, South	Cm	56.83	26.91
Godley	2002	Ascension Island, British Overseas Territory	Atlantic, South	Cm	75.40	26.91
Pintus	2009	Ascension Island, British Overseas Territory	Atlantic, South	Cm	91.00	26.39
Kaska	2006	Fethiye beach, Turkey	Mediterranean	Cc	62.75	26.95
Steckenreuter	2010	Huon Coast, Papua New Guinea	Pacific, West	Dc	19.07	28.77
Hanson	1998	Hutchinson Island, Florida	Atlantic, Northwest	Cc	100.00	28.22
Houghton	2001	Kefalonia, Greece	Mediterranean	Cc	59.20	24.90
Jribi	2014	Kuriat islands, Tunisia	Mediterranean	Cc	5.00	26.40
Rees	2004	Kyparissia Bay, Greece	Mediterranean	Cc	70.00	25.00

Hernández-Echeagaray	2012	La Escobilla, Mexico	Pacific, East	Lo	55.00	28.91
Jribi	2013	Sirte, Libya	Mediterranean	Cc	77.90	27.24
Oz	2004	Patara beach, Turkey	Mediterranean	Cc	70.50	26.95
Binckley	1998	Playa Grande, Costa Rica	Pacific, East	Dc	94.63	28.11
Sieg	2011	Playa Grande, Costa Rica	Pacific, East	Dc	83.00	28.05
Santidrián Tomillo	2014	Playa Grande, Costa Rica	Pacific, East	Dc	84.11	27.99
Rebelo	2012	Poilao, Guinea-Bissau	Atlantic, South	Cm	70.20	27.80
Bevan	2013	Rancho Nuevo, Mexico	Atlantic, Northwest	Lk	51.65	25.00
Laloë	2014	Sal, Cape Verde Islands	Atlantic, Northeast	Cc	81.78	26.38
Kilic	2014	Sugözü beaches, Turkey	Mediterranean	Cm	70.50	25.86
Hawkes	2007	Bald Head Island, USA	Atlantic, Northwest	Cc	58.00	27.11
LeBlanc	2012	Wassaw National Wildlife Refuge, USA	Atlantic, Northwest	Cc	61.05	26.69
Esteban	2016	Diego Garcia, Chagos Archipelago	Indian, Southwest	Ei	47.00	28.27
Esteban	2016	Diego Garcia, Chagos Archipelago	Indian, Southwest	Cm	37.00	28.27
Revuelta	2015	Jaragua National Park, Dominican Republic	Atlantic, Northwest	Dc	63.25	28.72
Schmid	2008	Keewaydin Island, Florida	Atlantic, Northwest	Cc	46.24	29.14
Mrosovsky	1984	Wia-Wia Nature Preserve, Suriname	Atlantic, North	Cm	53.90	28.10
Mrosovsky	1984	Wia-Wia Nature Preserve, Suriname	Atlantic, Northwest	Dc	49.00	28.10
Dutton	1985	Wia-Wia Nature Preserve, Suriname	Atlantic, Northwest	Dc	57.50	28.07
Garcia	2003	Playa Cuixmala, Jalisco	Pacific, East	Lo	72.50	28.93
Weston	2013	St. Croix, U.S. Virgin Islands	Atlantic, Northwest	Dc	89.60	28.57
Spotila	1987	Tortuguero, Costa Rica	Atlantic, North	Cm	50.38	30.00
Marcovaldi	2016	Sergipe, Brazil	Atlantic, Southwest	Cc	95.57	27.89
Marcovaldi	2016	Bahia, Brazil	Atlantic, Southwest	Cc	93.37	27.89
Marcovaldi	2016	Espirito Santo, Brazil	Atlantic, Southwest	Cc	58.87	27.24
Marcovaldi	2016	Rio de Janeiro, Brazil	Atlantic, Southwest	Cc	45.15	26.37

Kobayashi	2017	Kochi Beach, Japan	Pacific, North	Cc	60.00	25.93
Sari	2017	Goksu Delta, Turkey	Mediterranean	Cc	74.33	28.13
Sonmez	2016	Samandag Beach, Turkey	Mediterranean	Cm	80.26	29.00
Patrício	2013	Poilao Island, Africa	Atlantic, South	Cm	60.00	27.62
Candan	2016	Sugozu beaches, Turkey	Mediterranean	Cm	57.18	29.00
Rocha	2015	Santa Luzia Island, Cabo Verde archipelago	Atlantic, Northeast	Cc	66.85	26.72
Reneker	2016	Bald Head Island, USA	Atlantic, Northwest	Cc	61.42	27.08
Ozdilek	2016	Samandag Beach, Turkey	Mediterranean	Cm	76.35	26.92
Fuentes	2017	Praia de Forte, Brazil	Atlantic, Southwest	Cc	89.60	27.49
Casale	2000	Akyatan, Turkey	Mediterranean	Cm	92.40	25.99
Kaska	2005	Dalaman Beach, Turkey	Mediterranean	Cc	80.50	26.85
Fuller	2013	Cyprus	Mediterranean	Cc	89.00	26.97
Mrosovsky	1984	Barrier Islands of South Carolina and Georgia, USA	Atlantic, Northwest	Cc	56.30	27.56
Godfrey	1996	Matapica Canal, Suriname	Atlantic, North	Cm	63.80	28.16
Godfrey	1996	Matapica Canal, Suriname	Atlantic, Northwest	Dc	69.40	28.16
Basford	1988	Sandy Point, St. Croix	Atlantic, Northwest	Dc	89.80	28.97
Baskale	2005	Dalyan Beach, Turkey	Mediterranean	Cc	94.50	26.95
DeGregorio	2011	Bald Head Island, USA	Atlantic, Northwest	Cc	100.00	26.69
Stewart	2019	Redang Island, Malaysia	East Indian and Southeast Asia	Cm	93.00	29.23
Calderón-Peña	2020	Guanahacabibes Peninsula, Cuba	Atlantic, North	Cm	85.00	28.70
Tiwol	2001	Gulisaan Island, Malaysia	East Indian and Southeast Asia	Cm	70.00	28.56
Chan	1995	Rantau Abang, Malaysia	Pacific, West	Dc	97.75	29.28
Flores-Aguirre	2020	Yucatan Peninsula, Mexico	Atlantic, Northwest	Ei	79.94	28.13
Gross	1995	North Carolina, Florida	Atlantic, Northwest	Cc	39.29	27.90

Lolavar	2015	Boca Raton, Florida	Atlantic, Northwest	Cc	85.71	28.53
Maxwell	1988	Boteler Point, South Africa	Indian, Southwest	Cc	38.33	26.01
Mrosovsky	1982	Wia-Wia Nature Preserve, Suriname	Atlantic, Northwest	Cc	64.17	28.25
Patrício	2017	Guinea-Bissau, West Africa	Atlantic, South	Cm	53.90	27.49
Robledo-Avila	2022	Playa La Escobilla in Oaxaca, Mexico	Pacific, East	Lo	83.72	29.53
Sari	2016	Dalyan Beach, Turkey	Mediterranean	Cc	39.02	27.72
Simões	2014	praia de Merepe, Brazil	Atlantic, Southwest	Ei	86.53	27.51
Standora	1985	Sarawak Islands near Borne	East Indian and Southeast Asia	Cm	74.00	28.82
Standora	1985	Playa Nancite, Costa Rica	Pacific, East	Lo	31.63	28.57
Standora	1985	Tortuguero, Costa Rica	Atlantic, North	Cm	71.00	30.00
Tilley	2019	Ascension Island	Atlantic, South	Cm	71.87	27.73
Tolen	2021	Chagar Hutang beach, Redang Island	East Indian and Southeast Asia	Cm	52.00	29.74
Trono	1991	Baguan Island Marine Turtle Sanctuary	East Indian and Southeast Asia	Cm	88.37	28.62
Trono	1991	Baguan Island Marine Turtle Sanctuary	East Indian and Southeast Asia	Cm	96.55	28.62
Wibbels	1999	Buck Island, St Croix	Atlantic, Northwest	Ei	96.08	28.06
Wyneken	2015	Florida, USA	Atlantic, Northwest	Cc	88.36	28.37
Wyneken	2007	Beaches from North Carolina to Florida, USA	Atlantic, Northwest	Cc	73.16	28.47
Tanabe	2020	Red sea - Thuwal	Indian, Northwest	Ei	61.00	32.03
Tanabe	2020	Red sea - Al Lith	Indian, Northwest	Ei	97.00	31.94
Tanner	2019	The Republic of Cape Verde	Atlantic, Northeast	Cc	84.00	24.70
Meylan	2023	Bermuda	Atlantic, North	Cm	61.35	27.31
Staines	2023	Conflict Islands, Papua New Guinea	Pacific, Southwest	Cm	46.20	29.14
Staines	2023	Conflict Islands, Papua New Guinea	Pacific, Southwest	Ei	46.20	29.14
Laloë	2020	Tetiaroa, French Polynesia	South Central Pacific	Cm	46.00	27.79

Booth	2020	Raine Island, Australia	Pacific, Southwest	Cm	99.30	26.37
Jensen	2018	sGBR, Australia	Pacific, Southwest	Cm	68.00	26.55
Gammon	2023	Rosemary Island, Australia	Indian, Southeast	Nd	60.53	28.98
Read	2013	La Roche Percee, New Caledonia	Pacific, South	Cc	99.00	25.50
Read	2013	Mon Repos, Ausltralia	Pacific, South	Cc	70.00	25.66
Wiggins	2023	Long Beach, Ascension Island	Atlantic, South	Cm	70.00	27.67
Wiggins	2023	North East Bay, Ascension Island	Atlantic, South	Cm	95.50	27.67
Sari	2015	Goksu Delta, Turkey	Mediterranean	Cc	77.05	28.13
Tanabe	2020	Red sea - Small Global	Indian, Northwest	Ei	49.17	29.16
Tanabe	2020	Red sea - Wadi el Gemal	Indian, Northwest	Ei	97.00	31.05
Tanabe	2020	Red sea - Ras Baridi	Indian, Northwest	Ei	97.00	31.16
Tanabe	2020	Red sea - Small Global	Indian, Northwest	Cm	38.50	29.16
Tanabe	2020	Red sea - Wadi el Gemal	Indian, Northwest	Cm	92.00	31.05
Tanabe	2020	Red sea - Ras Baridi	Indian, Northwest	Cm	92.00	31.16
Tanabe	2020	Red sea - Thuwal	Indian, Northwest	Cm	60.00	32.03
Tanabe	2020	Red sea - Al Lith	Indian, Northwest	Cm	92.00	31.94
Castheloge	2018	Pirambu Beach, Brazil	Atlantic, West	Lo	95.00	27.91
DeOcampo	1998	Turtle Island, Phillippines	East Indian and Southeast Asia	Cm	100.00	28.41
Wright	2012	Alagadi beach, Northen Cyprus	Mediterranean	Cm	95.00	29.30
Tello-Sahagun	2023	Majahuas beach, Mexico	Pacific, East	Lo	40.25	29.11
Stubbs	2014	Cape Domett, WA	Indian, Southeast	Nd	83	28.69
Binhammer	2019	Playa Coyote, Costa Rica	Pacific, East	Lo	78.6	28.67
Valdez	2000	La Gloria, Mexico	Pacific, East	Lo	71.875	28.52

References

Basford, S. J. (1988). *Temperature Regimes on Sandy Point, St. Croix and Implications for Sex Determination of Hatchlings Leatherback Sea Turtles* (Order No. 13852896). Available from ProQuest One Academic. (2203396608). http://ezproxy.deakin.edu.au/login?url=https://www.proquest.com/dissertations-theses/temperature-regimes-on-sandy-point-st-croix/docview/2203396608/se-2

Başkale, E., & Kaska, Y. (2005). Sea turtle nest conservation techniques on southwestern beaches in Turkey. *Israel Journal of Ecology and Evolution*, *51*(1), 13-26.

Bevan, E. (2013). *Implications of hatchling sex ratios and survival in the recovery program for the endangered Kemp's ridley sea turtle* (Order No. 1543859). Available from ProQuest One Academic. (1433305582). http://ezproxy.deakin.edu.au/login?url=https://www.proquest.com/dissertations-theses/implications-hatchling-sex-ratios-survival/docview/1433305582/se-2

Binckley, C. A., Spotila, J. R., Wilson, K. S., & Paladino, F. V. (1998). Sex Determination and Sex Ratios of Pacific Leatherback Turtles, Dermochelys coriacea. *Copeia*, 1998(2), 291. https://doi.org/10.2307/1447425

Binhammer, M.R., Beange, M., Arauz, R. (2019). Sand Temperature, Sex Ratios, and Nest Success in Olive Ridley Sea Turtles. *Marine Turtle Newsletter*, 159:5-9, http://www.seaturtle.org/mtn/archives/mtn159/mtn159-2.shtml

Blamires, S., & Guinea, M. (2003). Emergence success of flatback sea turtles (Natator depressus) at Fog Bay, Northern Territory, Australia. *Chelonian Conservation and Biology*, *4*(3), 548-556.

Booth, D., Dunstan, A., Bell, I., Reina, R., & Tedeschi, J. (2020). Low male production at the world's largest green turtle rookery. Marine Ecology Progress Series, 653, 181–190. https://doi.org/10.3354/meps13500

Booth, D. T., & Freeman, C. (2006). Sand and nest temperatures and an estimate of hatchling sex ratio from the Heron Island green turtle (Chelonia mydas) rookery, Southern Great Barrier Reef. Coral Reefs, 25(4), 629–633. https://doi.org/10.1007/s00338-006-0135-4

Booth, D. T., & Astill, K. (2001). Temperature variation within and between nests of the green sea turtle, Chelonia mydas (Chelonia: Cheloniidae) on Heron Island, Great Barrier Reef. Australian Journal of Zoology, 49(1), 71. https://doi.org/10.1071/ZO00059

Broderick, A. C., Godley, B. J., & Hays, G. C. (2001). Metabolic Heating and the Prediction of Sex Ratios for Green Turtles (Chelonia mydas). Physiological and Biochemical Zoology, 74(2), 161–170. https://doi.org/10.1086/319661

Broderick, A., Godley, B., Reece, S., & Downie, J. (2000). Incubation periods and sex ratios of green turtles: Highly female biased hatchling production in the eastern Mediterranean. *Marine Ecology Progress Series*, 202, 273–281. https://doi.org/10.3354/meps202273

Calderón-Peña, R., Betancourt-Avila, R., Rodríguez-Fajardo, E., Martínez-González, Y., & Azanza Ricardo, J. (2020). Sex ratio of the green sea turtle Chelonia mydas (Testudines: Cheloniidae) hatchlings in the Guanahacabibes Peninsula, Cuba. *Revista de Biología Tropical, 68*(3). https://doi.org/10.15517/rbt.v68i3.39033

Candan, O., & Kolankaya, D. (2016). Sex Ratio of Green Turtle (Chelonia mydas) Hatchlings at Sugözü, Turkey: Higher Accuracy with Pivotal Incubation Duration. *Chelonian Conservation and Biology, 15*(1), 102–108. https://doi.org/10.2744/CCB-1132.1

Casale, P., Gerosa, G., & Yerli, S. V. (2000). Female-biased primary sex ratio of the Green Turtle, C helonia mydas , estimated through sand temperatures at Akyatan, Turkey. *Zoology in the Middle East, 20*(1), 37–46. https://doi.org/10.1080/09397140.2000.10637810

Castheloge, V.D. & Santos, Marcelo & Castilhos, J.C. & Filho, P.R.J. & Gomes, Levy & Clemente-Carvalho, Rute & Ferreira, Paulo. (2018). Pivotal temperature and hatchling sex ratio of olive ridley sea turtles lepidochelys olivaceafrom the south Atlantic Coast of Brazil. *Herpetological Conservation and Biology*, *13*, 488-496.

Chan, E. H., & Liew, H. C. (1995). Incubation temperatures and sex-ratios in the Malaysian leatherback turtle Dermochelys coriacea. *Biological Conservation, 74*(3), 169–174. https://doi.org/10.1016/0006-3207(95)00027-2

Chu, C.T., Booth, D.T., & Limpus, C.J. (2008). Estimating the sex ratio of loggerhead turtle hatchlings at Mon Repos rookery (Australia) from nest temperatures. *Australian Journal of Zoology*, *56*(1), 57-64. https://doi.org/10.1071/ZO08004

DeGregorio, B. A., & Williard, A. S. (2011). Incubation Temperatures and Metabolic Heating of Relocated and In Situ Loggerhead Sea Turtle (*Caretta caretta*) Nests at a Northern Rookery. *Chelonian Conservation and Biology, 10*(1), 54–61. https://doi.org/10.2744/CCB-0880.1

De Ocampo, G.D.D., Jaojoco, M., & Jaojoco, E.G. (1998). Incubation period, hatching percentage and sex ratio of green sea turtle (*Chelonia mydas L.*) hatchlings incubated at turtle islands, Philippines from April to June. *Philippine Journal of Veterinary Medicine*, *35*, 1 – 2. http://seaturtle.org/library/DeOcampoGD_1998_PhilJVetMed.pdf

Dutton, P. H., Whitmore, C. P., & Mrosovsky, N. (1985). Masculinisation of leatherback turtle Dermochelys coriacea hatchlings from eggs incubated in styrofoam boxes. *Biological Conservation, 31*(3), 249–264. https://doi.org/10.1016/0006-3207(85)90070-9

Esteban, N., Laloë, J.-O., Mortimer, J. A., Guzman, A. N., & Hays, G. C. (2016). Male hatchling production in sea turtles from one of the world's largest marine protected areas, the Chagos Archipelago. *Scientific Reports, 6*(1), 20339. https://doi.org/10.1038/srep20339

Flores-Aguirre, C. D., Díaz-Hernández, V., Ugarte, I. H. S., & Caballero, L. E. S. (2020). Feminization tendency of Hawksbill Turtles (Eretmochelys imbricata) in the western Yucatán Peninsula, Mexico. *Amphib. Reptile Conserv., 14*(1).

Fuentes, M. M. P. B., Monsinjon, J., Lopez, M., Lara, P., Santos, A., Dei Marcovaldi, M. A. G., & Girondot, M. (2017). Sex ratio estimates for species with temperature-dependent sex determination differ according to the proxy used. *Ecological Modelling, 365*, 55–67. https://doi.org/10.1016/j.ecolmodel.2017.09.022

Fuller, W., Godley, B., Hodgson, D., Reece, S., Witt, M., & Broderick, A. (2013). Importance of spatio-temporal data for predicting the effects of climate change on marine turtle sex ratios. *Marine Ecology Progress Series, 488*, 267–274. https://doi.org/10.3354/meps10419

Gammon, M. (2023). Characterising climate change vulnerability at flatback turtle nesting sites in the Pilbara region of Western Australia. *The University of Western Australia*. https://doi.org/10.26182/hde4-h382

Garcıía, A., Ceballos, G., & Adaya, R. (2003). Intensive beach management as an improved sea turtle conservation strategy in Mexico. *Biological Conservation, 111*(2), 253–261. https://doi.org/10.1016/S0006-3207(02)00300-2

Godfrey, M. H., D'Amato, A. F., Marcovaldi, M. Â., & Mrosovsky, N. (1999). Pivotal temperature and predicted sex ratios for hatchling hawksbill turtles from Brazil. *Canadian Journal of Zoology*, *77*(9), 1465-1473. https://doi.org/10.1139/z99-117

Godfrey, M. H., Mrosovsky, N., & Barreto, R. (1996). Estimating past and present sex ratios of sea turtles in Suriname. *Canadian Journal of Zoology, 74*(2), 267–277. https://doi.org/10.1139/z96-033

Godley, B., Broderick, A., Glen, F., & Hays, G. (2002). Temperature-dependent sex determination of Ascension Island green turtles. *Marine Ecology Progress Series, 226*, 115–124. https://doi.org/10.3354/meps226115

Godley, B. J., Broderick, A. C., & Mrosovsky, N. (2001). Estimating hatchling sex ratios of loggerhead turtles in Cyprus from incubation durations. *Marine Ecology Progress Series*, *210*, 195-201. http://dx.doi.org/10.3354/meps210195

Gross, T. S., Crain, D. A., Bjorndal, K. A., Bolten, A. B., & Carthy, R. R. (1995). Identification of sex in hatchling loggerhead turtles (Caretta caretta) by analysis of steroid concentrations in chorioallantoic/amniotic fluid. *General and comparative endocrinology*, *99*(2), 204-210. https://doi.org/10.1006/gcen.1995.1103

Hanson, J., Wibbels, T., & Martin, R. E. (1998). Predicted female bias in sex ratios of hatchling loggerhead sea turtles from a Florida nesting beach. *Canadian Journal of Zoology, 76*(10), 1850-1861. https://doi.org/10.1139/z98-118

Hawkes, L. A., Broderick, A. C., Godfrey, M. H., & Godley, B. J. (2007). Investigating the potential impacts of climate change on a marine turtle population. *Global Change Biology, 0*(0), 070621084512044-??? https://doi.org/10.1111/j.1385-2486.2006.01320.x

Hernández-Echeagaray, O. E., Hernández-Cornejo, R., Harfush-Meléndez, M., & García-Gasca, A. (2012). Evaluation of sex ratios of the olive ridley sea turtle (Lepidochelys olivacea) on the arribada nesting beach, La Escobilla, Mexico. *Marine Turtle Newsletter*, *133*, 12-16.

Houghton, J. D. R., & Hays, G. C. (2001). Asynchronous emergence by loggerhead turtle (Caretta caretta) hatchlings. *Naturwissenschaften*, *88*, 133-136. https://doi.org/10.1007/s001140100212

Ilgaz, Ç. E. T. İ. N., Özdemir, A., Kumlutaş, Y., & Durmuş, S. H. (2011). The effect of nest relocation on embryonic mortality and sex ratio of Loggerhead Turtles, Caretta caretta (Reptilia: Cheloniidae), at Dalyan Beach, Turkey. Italian Journal of Zoology, 78(3), 354–363. https://doi.org/10.1080/11250003.2010.509742

Jensen, M. P., Allen, C. D., Eguchi, T., Bell, I. P., LaCasella, E. L., Hilton, W. A., Hof, C. A. M., & Dutton, P. H. (2018). Environmental Warming and Feminization of One of the Largest Sea Turtle Populations in the World. *Current Biology, 28*(1), 154-159.e4. https://doi.org/10.1016/j.cub.2017.11.057

Jribi, I., & Bradai, M. N. (2014). Sex ratio estimations of loggerhead sea turtle hatchlings at Kuriat Islands, Tunisia: Can minor nesting sites contribute to compensate globally female-biased sex ratio?. *The Scientific World Journal*, *2014*(1), 419410. https://doi.org/10.1155/2014/419410

Jribi, I., Hamza, A., Saied, A., & Ouergui, A. (2013). Sex ratio estimations of loggerhead marine turtle hatchlings by incubation duration and nest temperature at Sirte beaches (Libya). *Scientia Marina, 77*(4), 617–624. https://doi.org/10.3989/scimar.03855.28B

Kaska, Y., Ilgaz, Ç., Özdemir, A., Başkale, E., Türkozan, O., Baran, İ., & Stachowitsch, M. (2006). Sex ratio estimations of loggerhead sea turtle hatchlings by histological examination and nest temperatures at Fethiye beach, Turkey. *Naturwissenschaften, 93*(7), 338–343. https://doi.org/10.1007/s00114-006-0110-5

Kaska, Y., Başkale, E., Katılmış, Y., & Urhan, R. (2005). Nest Temperatures and Sex Ratio Variations Among The Hatchlings And Embryos Of Loggerhead Turtles On Dalaman Beach, Turkey. *Proceedings, Second Mediterranean Conference on Marine Turtles, Kemer, 2005*

Kaska, Y., Downie, R., Tippett, R., & Furness, R. W. (1998). Natural temperature regimes for loggerhead and green turtle nests in the eastern Mediterranean. *Canadian journal of zoology*, *76*(4), 723-729. https://doi.org/10.1139/z97-245

Katselidis, K. A., Schofield, G., Stamou, G., Dimopoulos, P., & Pantis, J. D. (2012). Females first? Past, present and future variability in offspring sex ratio at a temperate sea turtle breeding area. *Animal Conservation, 15*(5), 508–518. https://doi.org/10.1111/j.1469-1795.2012.00543.x

Kılıç, Ç., & Candan, O. (2014). Hatchling sex ratio, body weight and nest parameters for Chelonia mydas nesting on Sugözü beaches (Turkey). *Animal Biodiversity and Conservation*, *37*(2), 177-182. https://doi.org/10.32800/abc.2014.37.0177

King, R., Cheng, W.-H., Tseng, C.-T., Chen, H., & Cheng, I.-J. (2013). Estimating the sex ratio of green sea turtles (Chelonia mydas) in Taiwan by the nest temperature and histological methods. *Journal of Experimental Marine Biology and Ecology, 445*, 140–147. https://doi.org/10.1016/j.jembe.2013.03.016

Kobayashi, S., Wada, M., Fujimoto, R., Kumazawa, Y., Arai, K., Watanabe, G., & Saito, T. (2017). The effects of nest incubation temperature on embryos and hatchlings of the loggerhead sea turtle: Implications of sex difference for survival rates during early life stages. *Journal of Experimental Marine Biology and Ecology, 486*, 274–281. https://doi.org/10.1016/j.jembe.2016.10.020

Laloë, J.-O., Monsinjon, J., Gaspar, C., Touron, M., Genet, Q., Stubbs, J., Girondot, M., & Hays, G. C. (2020). Production of male hatchlings at a remote South Pacific green sea turtle rookery: Conservation implications in a female-dominated world. *Marine Biology, 167*(5), 70. https://doi.org/10.1007/s00227-020-03686-x

Laloë, J.-O., Esteban, N., Berkel, J., & Hays, G. C. (2016). Sand temperatures for nesting sea turtles in the Caribbean: Implications for hatchling sex ratios in the face of climate

change. *Journal of Experimental Marine Biology and Ecology, 474*, 92–99. https://doi.org/10.1016/j.jembe.2015.09.015

Laloë, J.-O., Cozens, J., Renom, B., Taxonera, A., & Hays, G. C. (2014). Effects of rising temperature on the viability of an important sea turtle rookery. *Nature Climate Change, 4*(6), 513–518. https://doi.org/10.1038/nclimate2236

LeBlanc, A. M., Drake, K. K., Williams, K. L., Frick, M. G., Wibbels, T., & Rostal, D. C. (2012). Nest Temperatures and Hatchling Sex Ratios from Loggerhead Turtle Nests Incubated Under Natural Field Conditions in Georgia, United States. *Chelonian Conservation and Biology, 11*(1), 108–116. https://doi.org/10.2744/CCB-0915.1

Lolavar, A., & Wyneken, J. (2015). Effect of rainfall on loggerhead turtle nest temperatures, sand temperatures and hatchling sex. *Endangered Species Research, 28*(3), 235–247. https://doi.org/10.3354/esr00684

Loop, K.A., Miller, J.D., & Limpus, C.J. (1995). Nesting by the hawsbill turtle (*Eretmochelys imbricata*) on Milman Island, Great Barrier Reef, Australia. *Wildlife Research, (22)*, 241-251. https://doi.org/10.1071/WR9950241

Marcovaldi, M. A. G. dei, López-Mendilaharsu, M., Santos, A. S., Lopez, G. G., Godfrey, M. H., Tognin, F., Baptistotte, C., Thomé, J. C., Dias, A. C. C., De Castilhos, J. C., & Fuentes, M. M. P. B. (2016). Identification of loggerhead male producing beaches in the south Atlantic: Implications for conservation. *Journal of Experimental Marine Biology and Ecology, 477*, 14–22. https://doi.org/10.1016/j.jembe.2016.01.001

Dei Marcovaldi, M. A. G., Santos, A. J. B., Santos, A. S., Soares, L. S., Lopez, G. G., Godfrey, M. H., López-Mendilaharsu, M., & Fuentes, M. M. P. B. (2014). Spatio-temporal variation in the incubation duration and sex ratio of hawksbill hatchlings: Implication for future management. *Journal of Thermal Biology, 44*, 70–77. https://doi.org/10.1016/j.jtherbio.2014.06.010

Marcovaldi, M. Â., Godfrey, M. H., & Mrosovsky, N. (1997). Estimating sex ratios of loggerhead turtles in Brazil from pivotal incubation durations. *Canadian Journal of Zoology*, *75*(5), 755-770. https://doi.org/10.1139/z97-097

Maulany, R. I., Booth, D. T., & Baxter, G. S. (2012). Emergence Success and Sex Ratio of Natural and Relocated Nests of Olive Ridley Turtles from Alas Purwo National Park, East Java, Indonesia. *Copeia, 2012*(4), 738–747. https://doi.org/10.1643/CH-12-088

Maxwell, J. A., Motara, M. A., & Frank, G. H. (1988). A micro-environmental study of the effect of temperature on the sex ratios of the loggerhead turtle, Caretta caretta , from Tongaland, Natal. *South African Journal of Zoology, 23*(4), 342–350. https://doi.org/10.1080/02541858.1988.11448123

Meylan, A. B., Brost, B., Conrad, L. J., Denison, S. H., Flaherty, D. B., Gray, J. A., Hardy, R. F., Meylan, P. A., Schwenter, J. A., Tornwall, B., & Owens, D. W. (2024). Feminization of a mixed-stock foraging aggregation of immature green turtles (Chelonia mydas), 1975–2018. *Marine Biology, 171*(1), 11. https://doi.org/10.1007/s00227-023-04320-2

Mrosovsky, N., Bass, A., Corliss, L. A., Richardson, J. I., & Richardson, T. H. (1992). Pivotal and beach temperatures for hawksbill turtles nesting in Antigua. *Canadian Journal of Zoology, 70*(10), 1920–1925. https://doi.org/10.1139/z92-261

Mrosovsky, N. A. J. P., & Provancha, J. (1992). Sex ratio of hatchling loggerhead sea turtles: data and estimates from a 5-year study. *Canadian Journal of Zoology, 70*(3), 530-538. https://doi.org/10.1139/z92-080

Mrosovsky, N., & Provancha, J. (1989). Sex ratio of loggerhead sea turtles hatching on a Florida beach. *Canadian Journal of Zoology, 67*(10), 2533-2539. https://doi.org/10.1139/z89-358

Mrosovsky, N., Dutton, P. H., & Whitmore, C. P. (1984). Sex ratios of two species of sea turtle nesting in Suriname. *Canadian Journal of Zoology, 62*(11), 2227–2239. https://doi.org/10.1139/z84-324

Mrosovsky, N., Hopkins-Murphy, S. R., & Richardson, J. I. (1984). Sex ratio of sea turtles: seasonal changes. *Science, 225*(4663), 739-741. https://doi.org/10.1126/science.225.4663.739

Mrosovsky, N. (1982). Sex ratio bias in hatchling sea turtles from artificially incubated eggs. *Biological Conservation, 23*(4), 309–314. https://doi.org/10.1016/0006-3207(82)90087-8

Öz, M., Erdoğan, A., Kaska, Y., Düşen, S., Aslan, A. Z. İ. Z., Sert, H. A. K. A. N., Yavuz, M., & Tunc, M. R. (2004). Nest temperatures and sex-ratio estimates of loggerhead turtles at Patara beach on the southwestern coast of Turkey. *Canadian Journal of Zoology*, *82*(1), 94-101. http://dx.doi.org/10.1139/z03-200

Özdemir, A., Ilgaz, Ç., Durmuş, S. H., & Güçlü, Ö. (2011). The effect of the predicted air temperature change on incubation temperature, incubation duration, sex ratio and hatching success of loggerhead turtles. *Animal Biology*, *61*(4), 369-383. http://doi.org/10.1163/157075511x596864

Özdilek, Ş. Y., Sönmez, B. E. K. T. A. Ş., & Kaska, Y. (2016). Sex ratio estimations of Chelonia mydas hatchlings at Samandağ Beach, Turkey. *Turkish Journal of Zoology, 40*(4), 552-560. http://dx.doi.org/10.3906/zoo-1501-17

Patino-Martinez, J., Marco, A., Quiñones, L., & Hawkes, L. (2011). A potential tool to mitigate the impacts of climate change to the caribbean leatherback sea turtle. *Global Change Biology, 18*(2), 401–411. https://doi.org/10.1111/j.1385-2486.2011.02532.x

Patrício, A. (2013). Habitat Selection and Climate Change Impacts on Green Turtles from Poilão Island, Guinea-Bissau. *University of Exeter*.

https://ruffordorg.s3.amazonaws.com/media/project_reports/12317-1%20Detailed%20Final%20Report.pdf

Patrício, A., Marques, A., Barbosa, C., Broderick, A., Godley, B., Hawkes, L., Rebelo, R., Regalla, A., & Catry, P. (2017). Balanced primary sex ratios and resilience to climate change in a major sea turtle population. *Marine Ecology Progress Series, 577*, 189–203. https://doi.org/10.3354/meps12242

Perez, E. A., Marco, A., Martins, S., & Hawkes, L. A. (2016). Is this what a climate change-resilient population of marine turtles looks like?. *Biological Conservation*, *193*, 124-132. http://dx.doi.org/10.1016/j.biocon.2015.11.023

Pintus, K. J., Godley, B. J., McGowan, A., & Broderick, A. C. (2009). Impact of clutch relocation on green turtle offspring. *The Journal of wildlife management*, *73*(7), 1151-1157. https://doi.org/10.2193/2008-103

Read, T., Booth, D. T., & Limpus, C. J. (2013). Effect of nest temperature on hatchling phenotype of loggerhead turtles (Caretta caretta) from two South Pacific rookeries, Mon Repos and La Roche Percée. *Australian Journal of Zoology*, *60*(6), 402-411. https://doi.org/10.1071/ZO12079

Rebelo, R., Barbosa, C., Granadeiro, J. P., Indjai, B., Novais, B., Rosa, G. M., & Catry, P. (2012). Can leftovers from predators be reliably used to monitor marine turtle hatchling sex-ratios? The implications of prey selection by ghost crabs. *Marine Biology, 159*(3), 613–620. https://doi.org/10.1007/s00227-011-1839-8

Rees, A. F., & Margaritoulis, D. (2004). Beach temperatures, incubation durations and estimated hatchling sex ratio for loggerhead sea turtle nests in southern Kyparissia Bay, Greece. *Testudo*, *6*(1), 23-36.

Reneker, J. L., & Kamel, S. J. (2016). Climate change increases the production of female hatchlings at a northern sea turtle rookery. *Ecology, 97*(12), 3257-3264. https://doi.org/10.1002/ecy.1603

Revuelta, O., León, Y. M., Broderick, A. C., Feliz, P., Godley, B. J., Balbuena, J. A., Mason, A., Poulton, K., Savoré, S., Raga, J. A., & Tomás, J. (2015). Assessing the efficacy of direct conservation interventions: Clutch protection of the leatherback marine turtle in the Dominican Republic. *Oryx, 49*(4), 677–686. https://doi.org/10.1017/S0030605313001488

Robledo-Avila, L. A., Phillips-Farfán, B. V., Harfush Meléndez, M., Lopez Toledo, L., Tafolla Venegas, D., Herrera Vargas, Ma. A., Ruíz Cortés, D. V., & Meléndez-Herrera, E. (2022). Short communication: Ex-situ conservation in hatcheries is associated with spleen development in Lepidochelys olivacea turtle hatchlings. *Comparative Biochemistry and Physiology Part A: Molecular & Integrative Physiology, 265*, 111130. https://doi.org/10.1016/j.cbpa.2021.111130

Rocha, P. R., Melo, T., Rebelo, R., & Catry, P. (2015). A Significant Nesting Population of Loggerhead Turtles at the Nature Reserve of Santa Luzia, Cabo Verde. *Chelonian Conservation and Biology, 14*(2), 161–166. https://doi.org/10.2744/CCB-1143.1

Santidrián Tomillo, P., Oro, D., Paladino, F. V., Piedra, R., Sieg, A. E., & Spotila, J. R. (2014). High beach temperatures increased female-biased primary sex ratios but reduced output of female hatchlings in the leatherback turtle. *Biological Conservation, 176*, 71–79. https://doi.org/10.1016/j.biocon.2014.05.011

Sari, F., & Kaska, Y. (2017). Assessment of hatchery management for the loggerhead turtle (Caretta caretta) nests on Göksu Delta, Turkey. *Ocean & Coastal Management, 146*, 89–98. https://doi.org/10.1016/j.ocecoaman.2017.06.010

Sarı, F., & Kaska, Y. (2016). Histochemical and immunohistochemical studies of the gonads and paramesonephric ducts of male and female hatchlings of loggerhead sea

turtles (*Caretta caretta*). *Biotechnic & Histochemistry, 91*(6), 428–437. https://doi.org/10.1080/10520295.2016.1201143

Sarı, F., & Kaska, Y. (2015). Loggerhead sea turtle hatchling sex ratio differences between two nesting beaches in Turkey. *Israel Journal of Ecology and Evolution, 61*(3–4), 115–129. https://doi.org/10.1080/15659801.2015.1047681

Schmid, J. L., Addison, D. S., Donnelly, M. A., Shirley, M. A., & Wibbels, T. (2008). The Effect of Australian Pine (Casuarina equisetifolia) Removal on Loggerhead Sea Turtle (Caretta caretta) Incubation Temperatures on Keewaydin Island, Florida. *Journal of Coastal Research, 10055*, 214–220. https://doi.org/10.2112/SI55-001.1

Sieg, A., Binckley, C., Wallace, B., Tomillo, P., Reina, R., Paladino, F., & Spotila, J. (2011). Sex ratios of leatherback turtles: Hatchery translocation decreases metabolic heating and female bias. *Endangered Species Research, 15*(3), 195–204. https://doi.org/10.3354/esr00372

Simões, T. N., Silva, A. C. D., Santos, E. M. D., & Chagas, C. A. (2014). Temperatura de incubação e razão sexual em filhotes recém-eclodidos da tartaruga marinha Eretmochelys imbricata (Linnaeus, 1766) no município do Ipojuca, Pernambuco, Brasil. *Papéis Avulsos de Zoologia (São Paulo), 54*(25), 363–374. https://doi.org/10.1590/0031-1049.2014.54.25

Sönmez, B., Turan, C., Özdilek, Ş. Y., & Turan, F. (2016). Sex determination of green sea turtle (*Chelonia mydas*) hatchlings on the bases of morphological characters. *Journal of the Black Sea/Mediterranean Environment*

Spotila, J. R., Standora, E. A., Morreale, S. J., & Ruiz, G. J. (1987). Temperature dependent sex determination in the green turtle (Chelonia mydas): effects on the sex ratio on a natural nesting beach. *Herpetologica*, 74-81. https://www.jstor.org/stable/3892439

Staines, M. N., Versace, H., Laloë, J., Smith, C. E., Madden Hof, C. A., Booth, D. T., Tibbetts, I. R., & Hays, G. C. (2023). Short-term resilience to climate-induced temperature increases for equatorial sea turtle populations. *Global Change Biology, 29*(23), 6546–6557. https://doi.org/10.1111/gcb.16952

Standora, E. A., & Spotila, J. R. (1985). Temperature Dependent Sex Determination in Sea Turtles. Copeia, 1985(3), 711. https://doi.org/10.2307/1444765

Steckenreuter, A., Pilcher, N., Krüger, B., & Ben, J. (2010). Male-Biased Primary Sex Ratio of Leatherback Turtles (Dermochelys coriacea) at the Huon Coast, Papua New Guinea. *Chelonian Conservation and Biology, 9*(1), 123–128. https://doi.org/10.2744/CCB-0763.1

Stewart, T. A., Booth, D. T., & Rusli, M. U. (2019). Influence of sand grain size and nest microenvironment on incubation success, hatchling morphology and locomotion performance of green turtles (*Chelonia mydas*) at the Chagar Hutang Turtle Sanctuary, Redang Island, Malaysia. *Australian Journal of Zoology, 66*(6), 356. https://doi.org/10.1071/ZO19025

Stubbs, J. L., Kearney, M. R., Whiting, S. D., & Mitchell, N. J. (2014). Models of primary sex ratios at a major flatback turtle rookery show an anomalous masculinising trend. *Climate Change Responses, 1*(1), 3. https://doi.org/10.1186/s40665-014-0003-3

Tanabe, L. K., Ellis, J., Elsadek, I., & Berumen, M. L. (2020). Potential feminization of Red Sea turtle hatchlings as indicated by in situ sand temperature profiles. *Conservation Science and Practice, 2*(10), e266. https://doi.org/10.1111/csp2.266

Tanner, C., Marco, A., Martins, S., Abella-Perez, E., & Hawkes, L. (2019). Highly feminised sex-ratio estimations for the world’s third-largest nesting aggregation of loggerhead sea turtles. *Marine Ecology Progress Series, 621*, 209–219. https://doi.org/10.3354/meps12963

Tello-Sahagún, L. A., Ley-Quiñonez, C. P., Abreu-Grobois, F. A., Monsinjon, J. R., Zavala-Norzagaray, A. A., Girondot, M., & Hart, C. E. (2023). Neglecting cooler low-season nest protection could deprive sea turtle populations of valuable hatchlings. *Biological Conservation*, *277*, 109873. https://doi.org/10.1016/j.biocon.2022.109873

Tilley, D., Ball, S., Ellick, J., Godley, B. J., Weber, N., Weber, S. B., & Broderick, A. C. (2019). No evidence of fine scale thermal adaptation in green turtles. Journal of Experimental Marine Biology and Ecology, 514–515, 110–117. https://doi.org/10.1016/j.jembe.2019.04.001

Tiwol, J. M. (2001). Sex ratios of hatchlings of the green turtle, *Chelonia mydas*, in natural nesting grounds, in open beach hatchery, and in sheltered beach hatchery in Gulisaan Island, Saba. *University Malaysia Sabah*.

Tolen, N., Rusli, M. U., & Booth, D. T. (2021). Relocating green turtle (Chelonia mydas) eggs to open beach areas produces highly female-biased hatchlings. *Herpetological Conservation and Biology*, *16*(3), 639-651.

Trono, R. B. (1991). Philippine Marine Turtle Conservation Program. *Marine Turtle Newsletter, 53,* 5-7

Uçar, A. H., Kaska, Y., Ergene, S., Aymak, C., Kaçar, Y., Kaska, A., & İli, P. (2012). Sex Ratio Estimation of the Most Eastern Main Loggerhead Sea Turtle Nesting Site: Anamur Beach, Mersin, Turkey. *Israel Journal of Ecology & Evolution, 58*(1), 87–100. https://doi.org/10.1560/IJEE.58.1.87

González, C. V., Bátiz, F. S., & Vázquez, S. H. (2012). Proporción sexual en crías de la tortuga marina Lepidochelys olivacea, producida en corral de incubación en la playa de anidación La Gloria, Jalisco, México. *Boletín del Centro de Investigaciones Biológicas*, *34*(3).

Weston, E. G. (2013). Predicting leatherback sea turtle sex ratios using spatial interpolation of nesting beach temperatures. *The Charles E. Schmidt College of Science*

Wibbels, T., Hillis-Starr, Z. M., & Phillips, B. (1999). Female-biased sex ratios of hatchling hawksbill sea turtles from a Caribbean nesting beach. *Journal of Herpetology*, *33*(1), 142-144. https://doi.org/10.2307/1565556

Wiggins, J., Baum, D., Broderick, A. C., Capel, T., Colman, L. P., Hunt, T., Simmons, D. L., McGurk, J., Mortlock, L., Nightingale, R., Weber, N., & Weber, S. B. (2023). Efficacy of artificial nest shading as a climate change adaptation measure for marine turtles at Ascension Island. Wildlife Society Bulletin, 47(4), e1497. https://doi.org/10.1002/wsb.1497

Wood, A., Booth, D. T., & Limpus, C. J. (2014). Sun exposure, nest temperature and loggerhead turtle hatchlings: Implications for beach shading management strategies at sea turtle rookeries. *Journal of Experimental Marine Biology and Ecology, 451*, 105–114. https://doi.org/10.1016/j.jembe.2013.11.005

Wright, L. I., Stokes, K. L., Fuller, W. J., Godley, B. J., McGowan, A., Snape, R., ... & Broderick, A. C. (2012). Turtle mating patterns buffer against disruptive effects of climate change. *Proceedings of the Royal Society B: Biological Sciences*, *279*(1736), 2122-2127. https://doi.org/10.1098/rspb.2011.2285

Wyneken, J., & Lolavar, A. (2015). Loggerhead sea turtle environmental sex determination: Implications of moisture and temperature for climate change based predictions for species survival. *Journal of Experimental Zoology Part B: Molecular and Developmental Evolution, 324*(3), 295–314. https://doi.org/10.1002/jez.b.22620

Wyneken, J., Epperly, S. P., Crowder, L. B., Vaughan, J., & Blair Esper, K. (2007). Determining sex in posthatchling loggerhead sea turtles using multiple gonadal and

accessory duct characteristics. *Herpetologica*, *63*(1), 19-30. https://doi.org/10.1655/0018-0831(2007)63[19:DSIPLS]2.0.CO;2

Zbinden, J., Davey, C., Margaritoulis, D., & Arlettaz, R. (2007). Large spatial variation and female bias in the estimated sex ratio of loggerhead sea turtle hatchlings of a Mediterranean rookery. *Endangered Species Research, 3*, 305–312. https://doi.org/10.3354/esr00058
