# Supplementary material for: Local Adaptation May Help Mitigate Feminisation of Sea Turtle Populations Globally
Source: Glob Chang Biol. 2025 Aug 29;31(9):e70458. doi: 10.1111/gcb.70458 (PMC12396736; doi:10.1111/gcb.70458)
Supplement: Supplementary file 2 — Table S2: Sea turtle pivotal temperatures of key nesting sites around the globe with derived monthly air temperatures (ICOADS), representing the thermal conditions during the nesting period. Pivotal temperatures were either experimentally determined in a temperature‐controlled environment (laboratory) or calculated in situ (field study). [file GCB-31-e70458-s003.pdf]

Title: Local adaptation may help mitigate feminisation of sea turtle populations globally

Jared J. Tromp*, Melissa N. Staines, Jacques-Olivier Laloë and Graeme C. Hays
*Corresponding author E-mail: j.tromp@deakin.edu.au

Supplementary table 2

Table S2. Sea turtle pivotal temperatures of key nesting sites around the globe with derived monthly air temperatures (ICOADS), representing the thermal conditions during the nesting period. Pivotal temperatures were either experimentally determined in a temperature-controlled environment (laboratory) or calculated in-situ (field study).

First author	Year	Location	Species	Study type	Reported pivotal temperature	Pivotal temperature	Monthly air temperature
Maxwell	1988	South Africa	Cc	Field Study	28.5 - 29.6	29.05	26.32
Mrosovsky	2002	Kyparissia Bay, Greece	Cc	Laboratory	29.3	29.3	25.74
Broderick	2000	Northern Cyprus	Cm	Field Study	28.7,29.2	28.95	26.67
Matsuzawa	1998	Japan	Cc	Laboratory	29.7	29.7	26.73
Burgess	2006	southern Great Barrier Reef	Cm	Laboratory	28 - 30	29	26.82
Limpus	1985	Heron Island	Cc	Laboratory	28.7	28.7	26.82
Georges	1994	Australia	Cc	Laboratory	29	29	26.63
Limpus	1985	Mon Repos	Cc	Laboratory	27.7	27.7	26.63
Shaver	1988	Mexico	Lk	Laboratory	30.2	30.2	28.85
Bickney	1998	Costa Rica	Dc	Laboratory	29.4	29.4	28.22
McCoy	1983	Costa Rica	Lo	Laboratory	30	30	28.80
Wibbels	1998	Costa Rica	Lo	Laboratory	~31	31	28.80
Wen	2018	Costa Rica	Lo	Field Study	31.6	31.6	28.76
Spotila	1987	Costa Rica	Cm	Field Study	28.5 - 30.3	29.4	28.36
Yntema	1982	USA	Cc	Laboratory	30	30	27.59

Mrosovsky	1992	Antigua	Ei	Laboratory	29.2	29.2	28.36
Rimbolt-Baly	1987	Suriname and French Guiana	Dc	Laboratory	29 - 30	29.5	28.20
Rimbolt	1985	Suriname and French Guiana	Dc	Laboratory	28.75 - 29.75	29.25	28.20
Mrosovsky	1984	Suriname	Cm	Laboratory	28.8	28.8	28.44
Godfrey	2006	Suriname	Cm	Laboratory	29.2	29.2	28.39
Castheloge	2018	Brazil	Lo	Laboratory	30.7	30.7	27.98
Marcovaldi	1997	Brazil	Cc	Laboratory	29.2	29.2	27.82
Godfrey	1999	Brazil	Ei	Laboratory	29.6	29.6	27.82
Godley	2002	Ascension Island	Cm	Field Study	28.8	28.8	27.52
Mrosovsky	1988	USA - North Carolina	Cc	Laboratory	29,29.2	29.1	26.99
Mrosovsky	1988	USA - Georgia	Cc	Laboratory	Near but <28.5	28.5	27.59
Mrosovsky	1988	USA - Florida	Cc	Laboratory	29.2	29.2	28.64
Leh	1985	Sarawak	Cm	Field Study	<29.5	29.5	29.19
Miller	1981	Australia	Cm	Laboratory	<29.0	29	26.82
Standora	1985	Costa Rica	Cm	Field Study	28.0 - 30.3	29.15	28.36
Kaska	1998	Northern Cyprus & SW Turkey	Cm & Cc	Field Study	29	29	26.67
Chan	1995	Malaysia	Dc	Field Study	29.21 - 30.4	29.805	29.01
Dobbs	2010	Milman Island, Australia	Ei	Laboratory	29.2	29.2	28.89
Mohanty-Hejmadi	1986	Gahirmatha Marine Sanctuary, India	Lo	Laboratory	29.5	29.5	28.15
Tilley	2019	Ascension Island	Cm	Field Study	28.9	28.9	27.52
Tilley	2019	Ascension Island	Cm	Laboratory	29.75	29.75	27.52
Bently	2020	Cape Domett, Australia	Nd	Laboratory	29.6	29.6	27.15
Bently	2020	Eighty Mile Beach, Australia	Nd	Laboratory	31.1	31.1	29.59
Bently	2020	Thevenard Island, Australia	Nd	Laboratory	30.1	30.1	28.35
Bently	2020	Lacepede Islands, Australia	Cm	Laboratory	29.5	29.5	29.36
Bently	2020	Ningaloo, Australia	Cm	Laboratory	29.1	29.1	27.59
Howard	2015	Jardine River, Australia	Nd	Laboratory	30.4	30.4	28.93

Stubbs	2014	Cape Domett, Australia	Nd	Laboratory and field	29.4	29.4	29.26
Stubbs	2018	Ningaloo, Australia	Cm	Laboratory	29.2	29.2	27.59
King	2013	Lanyu Island, Taiwan	Cm	Field Study	29	29	29.02
LeBlanc	2012	Wassaw National Wildlife Refuge, USA	Cc	Field Study	28.9	28.9	27.55

References

Bentley, B. P., Stubbs, J. L., Whiting, S. D., & Mitchell, N. J. (2020). Variation in thermal traits describing sex determination and development in Western Australian sea turtle populations. *Functional Ecology*, *34*(11), 2302-2314. https://doi.org/10.1111/1365-2435.13645

Binckley, C. A., Spotila, J. R., Wilson, K. S., & Paladino, F. V. (1998). Sex determination and sex ratios of Pacific leatherback turtles, *Dermochelys coriacea*. *Copeia*, 291-300. https://doi.org/10.2307/1447425

Broderick, A. C., Godley, B. J., Reece, S., & Downie, J. R. (2000). Incubation periods and sex ratios of green turtles: highly female biased hatchling production in the eastern Mediterranean. *Marine Ecology Progress Series*, *202*, 273-281. http://dx.doi.org/10.3354/meps202273

Burgess, E. A., Booth, D. T., & Lanyon, J. M. (2006). Swimming performance of hatchling green turtles is affected by incubation temperature. *Coral reefs*, *25*, 341-349. https://doi.org/10.1007/s00338-006-0116-7

Castheloge, V.D., dos Santos, M.R.D.D., de Castilhos, J.C., Paulo Roberto de J. Filho, P.R.D.J., Gomes, L.D.C., Clemente-Carvalho, R.B.G., & Ferreira, P.D. (2018). Pivotal Temperature and Hatchling Sex Ratio of Olive Ridley Sea Turtles *Lepidochelys olivacea* from the South Atlantic Coast of Brazil. *Herpetological Conservation and Biology* *13*(2):488–496.

Chan, E. H., & Liew, H. C. (1995). Incubation temperatures and sex-ratios in the Malaysian leatherback turtle Dermochelys coriacea. *Biological conservation, 74*(3), 169-174. https://doi.org/10.1016/0006-3207(95)00027-2

Dobbs, K.A., Miller, J.D., Limpus, C., & Landry, A.M. (2010). Hawksbill Turtle Hatchling Sex Ratios and Incubation and Pivotal Temperatures From Milman Island, Great Barrier Reef, Australia. *Marine Turtle Newsletter 128*, 12-16

Georges, A., Limpus, C., & Stoutjesdijk, R. (1994). Hatchling sex in the marine turtle Caretta caretta is determined by proportion of development at a temperature, not daily duration of exposure. *Journal of Experimental Zoology*, *270*(5), 432-444. https://doi.org/10.1002/jez.1402700504

Godfrey, M. H., D'Amato, A. F., Marcovaldi, M. Â., & Mrosovsky, N. (1999). Pivotal temperature and predicted sex ratios for hatchling hawksbill turtles from Brazil. *Canadian Journal of Zoology*, *77*(9), 1465-1473. https://doi.org/10.1139/z99-117

Godfrey, M. H., & Mrosovsky, N. (2006). Pivotal temperature for green sea turtles, *Chelonia mydas*, nesting in Suriname. *The Herpetological Journal*, *16*(1), 55-61.

Godley, B. J., Broderick, A. C., Glen, F., & Hays, G. C. (2002). Temperature-dependent sex determination of Ascension Island green turtles. *Marine Ecology Progress Series*, *226*, 115-124. http://dx.doi.org/10.3354/meps226115

Howard, R., Bell, I., & Pike, D. A. (2015). Tropical flatback turtle (Natator depressus) embryos are resilient to the heat of climate change. *Journal of Experimental Biology*, *218*(20), 3330-3335. https://doi.org/10.1242/jeb.118778

Kaska, Y., Downie, R., Tippett, R., & Furness, R. W. (1998). Natural temperature regimes for loggerhead and green turtle nests in the eastern Mediterranean. *Canadian journal of zoology*, *76*(4), 723-729. https://doi.org/10.1139/z97-245

LeBlanc, A. M., Drake, K. K., Williams, K. L., Frick, M. G., Wibbels, T., & Rostal, D. C. (2012). Nest Temperatures and Hatchling Sex Ratios from Loggerhead Turtle Nests Incubated Under Natural Field Conditions in Georgia, United States. *Chelonian Conservation and Biology, 11*(1), 108–116. https://doi.org/10.2744/CCB-0915.1

Leh, C. M., Poon, S. K., & Siew, Y. C. (1985). Temperature-related phenomena affecting the sex of green turtle (Chelonia mydas) hatchlings in the Sarawak turtle islands. *Sarawak Museum Journal*, *34*(55), 183-193.

Limpus, C. J., Reed, P. C., & Miller, J. D. (1985). Temperature dependent sex determination in Queensland sea turtles: intraspecific variation in Caretta caretta. *Biology of Australasian frogs and reptiles*, *1985*, 343-351.

Marcovaldi, M. Â., Godfrey, M. H., & Mrosovsky, N. (1997). Estimating sex ratios of loggerhead turtles in Brazil from pivotal incubation durations. *Canadian Journal of Zoology*, *75*(5), 755-770. https://doi.org/10.1139/z97-097

Matsuzawa, Y., Sato, K., Tanaka, H., Bando, T., Sakamoto, W., & Gotou, K. (1998). Estimation of sex ratio of loggerhead turtles hatching on the Senri-coast in Japan. In *Proceedings of the Sixteenth Annual Symposium on Sea Turtle Biology and Conservation. NOAA Tech. Rep. NMFS-SEFSC-412* (pp. 101-102).

Maxwell, J. A., Motara, M. A., & Frank, G. H. (1988). A micro-environmental study of the effect of temperature on the sex ratios of the loggerhead turtle, *Caretta caretta*, from Tongaland, Natal. *South African Journal of Zoology*, *23*(4), 342–350. https://doi.org/10.1080/02541858.1988.11448123

McCoy, C. J., Vogt, R. C., & Censky, E. J. (1983). Temperature-controlled sex determination in the sea turtle Lepidochelys olivacea. *Journal of Herpetology*, *17*(4), 404-406. https://doi.org/10.2307/1563594

Miller, J. D., & Limpus, C. J. (1981). Incubation period and sexual differentiation in the green turtle *Chelonia mydas L*. In *Proceedings of the Melbourne Herpetological Symposium*, 66-73. The Zoological Board of Victoria: Melbourne

Mohanty-Hejmadi, P., Behra, M., & Dimond, M. T. (1985). Temperature dependent sex differentiation in the olive ridley *Lepidochelys olivacea* and its implications for conservation. In *Symposium on endangered marine animals and marine parks. Cochin. Marine Biological Association of India*,1-5

Mrosovsky, N., Kamel, S., Rees, A. F., & Margaritoulis, D. (2002). Pivotal temperature for loggerhead turtles (*Caretta caretta*) from Kyparissia Bay, Greece. *Canadian Journal of Zoology*, *80*(12), 2118-2124. https://doi.org/10.1139/z02-204

Mrosovsky, N. (1988). Pivotal temperatures for loggerhead turtles (*Caretta caretta*) from northern and southern nesting beaches. *Canadian Journal of Zoology*, *66*(3), 661-669. https://doi.org/10.1139/z88-098

Mrosovsky, N., Bass, A., Corliss, L. A., Richardson, J. I., & Richardson, T. H. (1992). Pivotal and beach temperatures for hawksbill turtles nesting in Antigua. *Canadian Journal of Zoology, 70*(10), 1920-1925. https://doi.org/10.1139/z92-261

Mrosovsky, N. (1988). Pivotal temperatures for loggerhead turtles (*Caretta caretta*) from northern and southern nesting beaches. *Canadian Journal of Zoology*, *66*(3), 661-669. https://doi.org/10.1139/z88-098

Mrosovsky, N., Dutton, P. H., & Whitmore, C. P. (1984). Sex ratios of two species of sea turtle nesting in Suriname. *Canadian Journal of Zoology*, *62*(11), 2227-2239. https://doi.org/10.1139/z84-324

Rimblot, F., Fretey, J., Mrosovsky, N., Lescure, J., & Pieau, C. (1985). Sexual differentiation as a function of the incubation temperature of eggs in the sea-turtle *Dermochelys coriacea* (Vandelli, 1761). *Amphibia-Reptilia, 6*(1), 83-92. https://doi.org/10.1163/156853885X00218

Rimblot, F., Fretey, J., Lescure, J., & Pieau, C. (1983, December). Influence de la temperature sur la differentiation sexuelle des gonades chez la tortue luth

(Dermochelys coriacea); etude en incubation artificielle et naturelle. In *Bases biologiques de l'aquaculture, Montpellier,* 12-16

Shaver, D. J., Owens, D. W., Chaney, A. H., Caillouet Jr, C. W., Burchfield, P., & Marquez, R. (1988). Styrofoam box and beach temperatures in relation to incubation and sex ratios of Kemp's ridley sea turtles. In *Schroeder BA (compiler):"The Eighth Annual Workshop on Sea Turtle Conservation and Biology." Fort Fisher, North Carolina: National Oceanic and Atmospheric Administration,* 103-108

Spotila, J. R., Standora, E. A., Morreale, S. J., & Ruiz, G. J. (1987). Temperature Dependent Sex Determination in the Green Turtle (*Chelonia mydas*): Effects on the Sex Ratio on a Natural Nesting Beach. *Herpetologica*, *43*(1), 74–81. http://www.jstor.org/stable/3892439

Standora, E. A., & Spotila, J. R. (1985). Temperature Dependent Sex Determination in Sea Turtles. *Copeia*, *1985*(3), 711–722. https://doi.org/10.2307/1444765

Stubbs, J. L., Kearney, M. R., Whiting, S. D., & Mitchell, N. J. (2014). Models of primary sex ratios at a major flatback turtle rookery show an anomalous masculinising trend. *Climate Change Responses*, *1*(1), 1-18. https://doi.org/10.1186/s40665-014-0003-3

Stubbs, J. L., & Mitchell, N. J. (2018). The influence of temperature on embryonic respiration, growth, and sex determination in a Western Australian population of green turtles (*Chelonia mydas*). *Physiological and Biochemical Zoology*, *91*(6), 1102-1114. https://doi.org/10.1086/700433

Tilley, D., Ball, S., Ellick, J., Godley, B. J., Weber, N., Weber, S. B., & Broderick, A. C. (2019). No evidence of fine scale thermal adaptation in green turtles. *Journal of Experimental Marine Biology and Ecology*, *514-515*, 110-117. https://doi.org/10.1016/j.jembe.2019.04.001

Wen, F. (2018). Field Pivotal Temperature and Hatchling Sex Ratios of Olive Ridley Sea Turtles (*Lepidochelys olivacea*) at Ostional Beach, Costa Rica (Order No. 10843448). Available from ProQuest One Academic. (2088909558). http://ezproxy.deakin.edu.au/login?url=https://www.proquest.com/dissertations-theses/field-pivotal-temperature-hatchling-sex-ratios/docview/2088909558/se-2

Wibbels, T., Rostal, D., & Byles, R. (1998). High Pivotal Temperature in the Sex Determination of the Olive Ridley Sea Turtle, *Lepidochelys olivacea*, from Playa Nancite, Costa Rica. *Copeia*, *1998*(4), 1086–1088. https://doi.org/10.2307/1447364

Yntema, C. L., & Mrosovsky, N. (1982). Critical periods and pivotal temperatures for sexual differentiation in loggerhead sea turtles. *Canadian Journal of Zoology*, *60*(5), 1012-1016. https://doi.org/10.1139/z82-141
