# Supplementary material for: Local Adaptation May Help Mitigate Feminisation of Sea Turtle Populations Globally
Source: Glob Chang Biol. 2025 Aug 29;31(9):e70458. doi: 10.1111/gcb.70458 (PMC12396736; doi:10.1111/gcb.70458)
Supplement: Supplementary file 3 — Table S3: Sea turtle nesting season of key nesting sites around the globe. Months highlighted in bold indicate the peak of nesting season as given in the reference. [file GCB-31-e70458-s001.pdf]

Title: Local adaptation may help mitigate feminisation of sea turtle populations globally

Jared J. Tromp*, Melissa N. Staines, Jacques-Olivier Laloë and Graeme C. Hays
*Corresponding author E-mail: j.tromp@deakin.edu.au

Supplementary table 3

Table S3: Sea turtle nesting season of key nesting sites around the globe. Months highlighted in bold indicate the peak of nesting season as given in the reference.

		Month											
**Site**	**Species**	**J**	**F**	**M**	**A**	**M**	**J**	**J**	**A**	**S**	**O**	**N**	**D**
Barrow Island, Australia[1]	Flatback										O	N	D
Cape Domett, Australia[2]	Flatback				A	M	J	J	A	S	O	N	
Cape Domett, Australia[3]	Flatback								A	S			
Cemetery Beach, Australia[1]	Flatback										O	N	D
Eighty Mile Beach, Australia[3]	Flatback											N	D
Fog bay, Australia[4]	Flatback			M	A	M	J	J	A	S	O	N	
Jardine River, Australia[5]	Flatback	J							A	S	O	N	D
Mundabullangana, Australia[1]	Flatback										O	N	D
Rosemary Island, Australia[6]	Flatback	J	F									N	D
Thevenard Island, Australia[3]	Flatback											N	D
Ascension Island, British Overseas Territory[7]	Green	J	F	M	A	M							
Ascension Island, British Overseas Territory[8]	Green	J	F	M	A	M							
Ascension Island, British Overseas Territory[9]	Green	J	F	M	A	M							
Ascension Island, British Overseas Territory[10]	Green	J	F	M	A	M							
Ascension Island, British Overseas Territory[11]	Green	J	F	M	A	M							

Long Beach, Ascension Island[12]	Green	J	F	M	A	M							
North East Bay, Ascension Island[12]	Green	J	F	M	A	M							
Alagadi, North Cyprus[13]	Green					M	J	J	A	S	O		
Akdeniz, Cyprus[15]	Green						J	J					
Akyatan, Turkey[16]	Green					M	J	J	A				
Alagadi, North Cyprus[14]	Green					M	J	J	A	S	O		
Bioko Island, Equatorial Guinnea[17]	Green	J	F									N	D
Baguan Island Marine Turtle Sanctuary[18]	Green							J	A	S	O	N	D
Bermuda[19]	Green				A	M	J	J	A	S	O		
Chagar Hutang beach, Redang Island[20]	Green					M	**J**	**J**	**A**	S			
Conflict Islands, Papua New Guinea[21]	Green	J	F	M								N	D
Costa Rica[22]	Green			M	A	M	J	J	A	S	O		
Costa Rica[23]	Green			M	A	M	J	J	A	S	O		
Diego Garcia, Chagos Archipelago[24]	Green	J	F	M	A	M	J	J	**A**	**S**	**O**	N	D
Guanahacabibes Peninsula, Cuba[25]	Green							**J**					
Guinea-Bissau, West Africa[26]	Green						J	J	**A**	**S**	O	N	D
Gulisaan Island, Malaysia[27]	Green	J										N	D
Heron Island, Australia[28]	Green	J	F	M	A						O	N	D
Heron Island, Australia[29]	Green	J	F	M	A						O	N	D
Heron Island, Australia[30]	Green	J	F	M	A						O	N	D
Lacepede Islands, Australia[3]	Green	J											D
Lanyu Island, Taiwan[31]	Green						J	J	A	S	O		
Matapica Canal, Suriname[32]	Green		F	M	A	M	J	J	A	S			

Moheli, Comoros[33]	Green			M	A	M	J	J	A				
nGBR, Australia[34]	Green	J	F	M								N	D
Nicobar Islands, India[35]	Green					M	J	J	A	S			
Ningaloo, Australia[3]	Green	J											D
Ningaloo, Australia[36]	Green	J											D
Poilao Island, Africa[37]	Green					M	J	J	A	S	O		
Poilao, Guinea-Bissau[38]	Green							J	A	S	O	N	
Raine Island, Australia[39]	Green	J	F	M	A						O	N	D
Red sea - Small Global[40]	Green					M	J	J	A	S			
Red sea - Al Lith[40]	Green					M	J	J	A	S			
Red sea - Ras Baridi[40]	Green								A	S	O	N	
Red sea - Thuwal[40]	Green					M	J	J	A	S			
Red sea - Wadi el Gemal[40]	Green					M	J	J	A	S			
Redang Island, Malaysia[41]	Green					M	J	J	A	S			
Samandag Beach, Turkey[43]	Green						J	J	A	S			
Samandag Beach, Turkey[44]	Green						J	J	A	S			
Sarawak Islands near Borne[45]	Green								A	S	O		
Sarawak Islands near Borne[23]	Green					M	J	J	A	S			
sGBR, Australia[34]	Green	J	F	M	A						O	N	D
southern Great Barrier Reef, Australia[46]	Green	J	F	M	A						O	N	D
St Eustatius, Dutch Caribbean[42]	Green				A	M	J	J	A	S	O		
Sugözü beaches, Turkey[47]	Green						J	J	A	S			
Sugozu beaches, Turkey[48]	Green						J	J	**A**	**S**			

Suriname[50]	Green		F	M	A	M	J	J	A				
Tetiaroa, French Polynesia[51]	Green	J	F	M	A				A	S	O	N	D
Tortuguero, Costa Rica[22]	Green			M	A	M	J	J	A	S	O		
Tortuguero, Costa Rica[23]	Green			M	A	M	J	J	A	S	O		
Tortuguero, Costa Rica[52]	Green			M	A	M	J	J	A	S	O		
Turtle Island, Phillippines[53]	Green	J	F	M	A	M	J	J	**A**	**S**	**O**	N	D
Wia-Wia Nature Preserve, Suriname[49]	Green		F	M	**A**	M	J	J	A				
Antigua[54]	Hawksbill						J	J	**A**	**S**	**O**	N	
Bahia, Brazil[55]	Hawksbill	J	F	M						S	O	N	D
Bahia, Brazil[56]	Hawksbill	J	F	M	A						O	N	D
Bioko Island, Equatorial Guinnea[17]	Hawksbill	J	F	M									D
Buck Island, St Croix[57]	Hawksbill			M	A	M	J	J	A	S	O		
Conflict Islands, Papua New Guinea[21]	Hawksbill	J	F	M								N	D
Curieuse Island, Seychelles[58]	Hawksbill	J	F	M	A					S	O	N	D
Diego Garcia, Chagos Archipelago[24]	Hawksbill	**J**	**F**	M	A	M	J			S	O	**N**	**D**
Milman Island, Australia[59]	Hawksbill	J	F	M									
Milman Island, Australia[60]	Hawksbill	J	F	M	A							N	D
Nicobar Islands, India[35]	Hawksbill							J	A	S	O	N	D
Pasture Bay, Antigua[61]	Hawksbill						J	J	A	S	O	N	
praia de Merepe, Brazil[62]	Hawksbill	J	F	M	A	M	J						
Red sea - Al Lith[40]	Hawksbill					M	J	J	A	S			
Red sea - Ras Baridi[40]	Hawksbill								A	S	O	N	
Red sea - Small Global[40]	Hawksbill					M	J	J	A	S			

Red sea - Thuwal[40]	Hawksbill					M	J	J	A	S			
Red sea - Wadi el Gemal[40]	Hawksbill					M	J	J	A	S			
Rio Grade do Norte, Brazil[40]	Hawksbill	J	F	M	A						O	N	D
St Eustatius, Dutch Caribbean[42]	Hawksbill	J	F		A	M	J	J	A	S	O	N	D
Yucatan Peninsula, Mexico[63]	Hawksbill						J	J	A	S			
Mexico[64]	Kemp's ridley				A	M	J	J	A				
Padre Island, USA[65]	Kemp's ridley				A	M	J	J					
Rancho Nuevo, Mexico[66]	Kemp's ridley			M	A	M	J	J	A				
Bioko Island, Equatorial Guinnea[17]	Leatherback	J	F									N	D
Bocas del Toro, Panama[67]	Leatherback		F	M	A	M	J	J	A	S	O	N	
Capitancito, Columbia[68]	Leatherback		F	M	A	M	J						
Huon Coast, Papua New Guinea[70]	Leatherback	**J**	F	M							O	N	**D**
Jamursba-Medi, Indonesia[71]	Leatherback			M	A	M	J	J	A	S	O	N	
Jaragua National Park, Dominican Republic[72]	Leatherback			M	A	M	J	J	A				
Matapica Canal, Suriname[32]	Leatherback			M	A	M	J	J	A	S			
Mayumba National Park, Gabon[71]	Leatherback	J	F	M								N	D
Nicobar Islands, India[35]	Leatherback	J	F	M							O	N	D
Pacuare, Costa Rica[73]	Leatherback			M	A	M	J	J	A	S			
Playa Grande, Costa Rica[69]	Leatherback	J	F									N	**D**
Playa Grande, Costa Rica[74]	Leatherback	J	F								O	N	D
Playa Grande, Costa Rica[75]	Leatherback	J	F	M							O	N	D
Playa Grande, Costa Rica[76]	Leatherback	J	F	M							O	N	D

Point Denis, Gabon[77]	Leatherback	J	F	M								N	D
Rantau Abang, Malaysia[78]	Leatherback			M	A	M	J	J	A	S	O		
Sandy Point, St. Croix[79]	Leatherback				A	M	J	J	A				
Sandy Point, USA[76]	Leatherback			M	A	M	J	J	A				
St Eustatius, Dutch Caribbean[42]	Leatherback		F	M	A	M	J	J					
St. Croix, U.S. Virgin Islands[80]	Leatherback					M	J	J	A				
Suriname and French Guiana[81]	Leatherback				A	M	J	J	A				
Suriname and French Guiana[82]	Leatherback				A	M	J	J	A				
Tortuguero, Costa Rica[76]	Leatherback			M	A	M	J						
Wia-Wia Nature Preserve, Suriname[49]	Leatherback		F	M	A	**M**	J	J	A				
Wia-Wia Nature Preserve, Suriname[83]	Leatherback				A	M	J	J	A				
Akdeniz, Cyprus[15]	Loggerhead						J	J					
Alagadi, Cyprus[84]	Loggerhead					M	J	J	A				
Alagadi, North Cyprus[85]	Loggerhead					M	J	J	A	S	O		
Anamur, Turkey[86]	Loggerhead						**J**	**J**	A	S			
Arabian sea coast, Oman[87]	Loggerhead				A	M	J	J	A	S			
Australia[88]	Loggerhead	J										N	D
Bahia, Brazil[89]	Loggerhead	J	F	M						S	O	N	D
Bahia, Brazil[90]	Loggerhead	J	F	M						S	O	**N**	**D**
Bald Head Island, USA[91]	Loggerhead					M	J	J	A	S	O		
Bald Head Island, USA[92]	Loggerhead					M	J	J	A				
Bald Head Island, USA[93]	Loggerhead					M	J	J	A	S	O		
Barrier Islands of South Carolina and Georgia, USA[94]	Loggerhead					M	J	J	A				

Beaches from North Carolina to Florida, USA[95]	Loggerhead				A	M	J	J	A				
Boa Vista, Cape Verde Islands[96]	Loggerhead						J	J	A	S	O		
Boa Vista, Cape Verde[97]	Loggerhead						J	J	A	S	O		
Boca Raton, Florida[98]	Loggerhead				A	M	J	J	A	S	O		
Boteler Point, South Africa[99]	Loggerhead	J									O	N	D
Cape Canaveral Air Force Station, Florida[100]	Loggerhead					M	J	J	A	S			
Cape Canaveral Air Force Station, Florida[101]	Loggerhead					M	J	J	A	S			
Cay Sal Bank, Bahamas[102]	Loggerhead				A	M	J	J	A				
Cayos de San Felipe, Cuba[84]	Loggerhead				A	M	J	J	A				
Cyprus[103]	Loggerhead					M	J	J	A	S	O		
Dalaman Beach, Turkey[104]	Loggerhead					M	J	J	A				
Dalyan Beach, Turkey[105]	Loggerhead						J	J	A	S			
Dalyan Beach, Turkey[106]	Loggerhead					M	J	J	A				
Dalyan, Turkey[107]	Loggerhead					M	J	J	A	S			
Dalyan, Turkey[108]	Loggerhead					M	J	J	A	S			
Dalyan, Turkey[109]	Loggerhead					M	J	J	A	S			
Dirk Hartog Island, Australia[110]	Loggerhead	J	F	M	A							N	D
Espirito Santo, Brazil[89]	Loggerhead	J	F	M						S	O	N	D
Espirito Santo, Brazil[90]	Loggerhead	J	F	M						S	O	**N**	**D**
Espírito Santo, Brazil[84]	Loggerhead	J	F	M						S	O	N	D
Fethiye beach, Turkey[111]	Loggerhead					M	**J**	**J**	A				
Fethiye, Turkey[84]	Loggerhead					M	J	J	A				
Florida, USA[112]	Loggerhead				A	M	J	J	A				

Florida, USA[113]	Loggerhead				A	M	J	J	A	S			
Florida, USA[114]	Loggerhead				A	M	J	J	A	S			
Forteith, Libya[84]	Loggerhead					M	J	J	A				
Georgia, USA[113]	Loggerhead					M	J	J	A				
Georgia, USA[115]	Loggerhead					M	J	J	A				
Gnaraloo Bay, Australia[116]	Loggerhead	J	F									N	D
Goksu Delta, Turkey[117]	Loggerhead					M	J	J	A	S			
Goksu Delta, Turkey[107]	Loggerhead					M	J	J	A	S			
Halaniyat Islands, Oman[87]	Loggerhead				A	M	J	J	A	S			
Heron Island[118]	Loggerhead	J	F	M							O	N	D
Hutchinson Island, Florida[119]	Loggerhead				A	M	**J**	**J**	A	S			
Japan[120]	Loggerhead				A	M	J	J	A				
Keewaydin Island, Florida[121]	Loggerhead					M	J	J	A				
Kefalonia, Greece[122]	Loggerhead					M	J	J	A	S			
Kochi Beach, Japan[123]	Loggerhead					M	J	J					
Kuriat islands, Tunisia[124]	Loggerhead						J	**J**	A				
KwaZulu-Natal, South Africa[84]	Loggerhead	J	F	M							O	N	D
Kyparissia Bay, Greece[125]	Loggerhead					M	J	J	A	S	O		
Kyparissia Bay, Greece[126]	Loggerhead					M	J	J	A	S	O		
La Roche Percee, New Caledonia[127]	Loggerhead											N	D
Maputo, Mozambique[84]	Loggerhead	J	F	M	A						O	N	D
Masirah Island, Oman[115]	Loggerhead				A	M	J	J	A	S			
Mediterranean shore, Israel[84]	Loggerhead					M	J	J	A				

Minabe-Senri, Japan[115]	Loggerhead				A	M	J	J	A				
Mon Repos[118]	Loggerhead	J	F	M							O	N	D
Mon Repos, Ausltralia[128]	Loggerhead	J	F	M							O	N	D
Mon Repos, Ausltralia[129]	Loggerhead	J	F	M								N	D
Mon Repos, Ausltralia[127]	Loggerhead	J											
North Carolina, Florida[130]	Loggerhead				A	M	J	J	A	S			
North Carolina, USA[113]	Loggerhead				A	M	J	J	A	S			
North Carolina, USA[84]	Loggerhead				A	M	J	J	A	S			
Patara beach, Turkey[131]	Loggerhead					M	J	J	A				
Praia de Forte, Brazil[132]	Loggerhead	J	F	M							O	N	D
Queensland, Australia[133]	Loggerhead	J	F	M							O	N	D
Quintana Roo, Mexico[84]	Loggerhead				A	M	J	J	A				
Rio de Janeiro, Brazil[90]	Loggerhead	J	F	M						S	O	**N**	**D**
Rio Longa, Angola[84]	Loggerhead	J	F								O	N	D
Roche Percée, New Caledonia[134]	Loggerhead	J										N	D
Sal, Cape Verde Islands[135]	Loggerhead						J	J	A	S	O	N	
Sal, Cape Verde[136]	Loggerhead						J	J	A	S	O	N	
Santa Luzia Island, Cabo Verde archipelago[137]	Loggerhead							J	A	S	O		
Sergipe, Brazil[89]	Loggerhead	J	F	M						S	O	N	D
Sergipe, Brazil[90]	Loggerhead	J	F	M						S	O	**N**	**D**
Sirte, Libya[138]	Loggerhead					M	J	J	A	S			
South Carolina, USA[115]	Loggerhead					M	J	J	A				
South Murion Island, Australia[87]	Loggerhead	J	F	M	A							N	D

The Republic of Cape Verde[139]	Loggerhead						J	J	A	S	O	N	
USA[140]	Loggerhead					M	J	J	A				
Wassaw National Wildlife Refuge, USA[141]	Loggerhead					M	J	J	A				
Wia-Wia Nature Preserve, Suriname[142]	Loggerhead					M	J						
Zakynthos island, Greece[143]	Loggerhead					M	J	J	A	S	O		
Zakynthos island, Greece[144]	Loggerhead					M	J	J	A	S	O		
Zakynthos, Greece[84]	Loggerhead					M	J	J	A	S			
Alas Purwo National Park, Indonesia[145]	Olive ridley					**M**	**J**	**J**					
Bioko Island, Equatorial Guinnea[17]	Olive ridley	J	F									N	D
Costa Rica[147]	Olive ridley						J	J	A	S	O		
Costa Rica[148]	Olive ridley						J	J	A	S	O		
Costa Rica[149]	Olive ridley	J							A	S	O	N	D
Devi Mouth, India[150]	Olive ridley		F	M									
Gahirmatha Marine Sanctuary, India[151]	Olive ridley		F	M									
Gahirmatha, India[150]	Olive ridley		F	M									
La Escobilla, Mexico[152]	Olive ridley	J	F	M	A	M	J	J	A	S	O	N	D
La Gloria, Mexico[153]	Olive ridley								A	S	O	N	D
Majahuas beach, Mexico[154]	Olive ridley	J	F	M	A	M	J	J	A	S	O	N	D
Nicobar Islands, India[35]	Olive ridley	J	F	M	A							N	D
Pirambu Beach, Brazil[146]	Olive ridley	J	F	M									
Playa Coyote, Costa Rica[155]	Olive ridley	J							A	S	O	N	D
Playa Cuixmala, Jalisco[156]	Olive ridley	J	F	M				J	A	S	O	N	D
Playa La Escobilla in Oaxaca, Mexico[157]	Olive ridley	J	F	M	A	M	J	J	A	S	O	N	D

Playa Nancite, Costa Rica[23]	Olive ridley	J							A	S	O	N	D
Point Denis, Gabon[77]	Olive ridley	J	F	M						S	O	N	D
Rushikulya, India[150]	Olive ridley		F	M									

References

1. Pendoley, K. L., Bell, C. D., McCracken, R., Ball, K. R., Sherborne, J., Oates, J. E., Becker, P., Vitenbergs, A., & Whittock, P. A. (2014). Reproductive biology of the flatback turtle Natator depressus in Western Australia. *Endangered Species Research*, *23*(2), 115-123.
2. Stubbs, J. L., Kearney, M. R., Whiting, S. D., & Mitchell, N. J. (2014). Models of primary sex ratios at a major flatback turtle rookery show an anomalous masculinising trend. *Climate Change Responses, 1*(1), 3. https://doi.org/10.1186/s40665-014-0003-3
3. Bentley, B. P., Stubbs, J. L., Whiting, S. D., & Mitchell, N. J. (2020). Variation in thermal traits describing sex determination and development in Western Australian sea turtle populations. *Functional Ecology*, *34*(11), 2302-2314. https://doi.org/10.1111/1365-2435.13645
4. Blamires, S., & Guinea, M. (2003). Emergence success of flatback sea turtles (Natator depressus) at Fog Bay, Northern Territory, Australia. *Chelonian Conservation and Biology*, *4*(3), 548-556.
5. Howard, R., Bell, I., & Pike, D. A. (2015). Tropical flatback turtle (Natator depressus) embryos are resilient to the heat of climate change. *Journal of Experimental Biology*, *218*(20), 3330-3335. https://doi.org/10.1242/jeb.118778
6. Gammon, M. (2023). Characterising climate change vulnerability at flatback turtle nesting sites in the Pilbara region of Western Australia. *The University of Western Australia*. https://doi.org/10.26182/hde4-h382
7. Godley, B. J., Broderick, A. C., & Hays, G. C. (2001). Nesting of green turtles (chelonia mydas) at Ascension Island, South Atlantic. *Biological Conservation*, *97*(2), 151-158.
8. Broderick, A. C., Godley, B. J., & Hays, G. C. (2001). Metabolic Heating and the Prediction of Sex Ratios for Green Turtles (Chelonia mydas). Physiological and Biochemical Zoology, 74(2), 161–170. https://doi.org/10.1086/319661

9. Godley, B., Broderick, A., Glen, F., & Hays, G. (2002). Temperature-dependent sex determination of Ascension Island green turtles. *Marine Ecology Progress Series, 226*, 115–124. https://doi.org/10.3354/meps226115
10. Pintus, K. J., Godley, B. J., McGowan, A., & Broderick, A. C. (2009). Impact of clutch relocation on green turtle offspring. *The Journal of wildlife management*, *73*(7), 1151-1157. https://doi.org/10.2193/2008-103
11. Tilley, D., Ball, S., Ellick, J., Godley, B. J., Weber, N., Weber, S. B., & Broderick, A. C. (2019). No evidence of fine scale thermal adaptation in green turtles. Journal of Experimental Marine Biology and Ecology, 514–515, 110–117. https://doi.org/10.1016/j.jembe.2019.04.001
12. Wiggins, J., Baum, D., Broderick, A. C., Capel, T., Colman, L. P., Hunt, T., Simmons, D. L., McGurk, J., Mortlock, L., Nightingale, R., Weber, N., & Weber, S. B. (2023). Efficacy of artificial nest shading as a climate change adaptation measure for marine turtles at Ascension Island. Wildlife Society Bulletin, 47(4), e1497. https://doi.org/10.1002/wsb.1497
13. Broderick, A., Godley, B., Reece, S., & Downie, J. (2000). Incubation periods and sex ratios of green turtles: Highly female biased hatchling production in the eastern Mediterranean. *Marine Ecology Progress Series*, 202, 273–281. https://doi.org/10.3354/meps202273
14. Wright, L. I., Stokes, K. L., Fuller, W. J., Godley, B. J., McGowan, A., Snape, R., ... & Broderick, A. C. (2012). Turtle mating patterns buffer against disruptive effects of climate change. *Proceedings of the Royal Society B: Biological Sciences*, *279*(1736), 2122-2127. https://doi.org/10.1098/rspb.2011.2285
15. Kaska, Y., Downie, R., Tippett, R., & Furness, R. W. (1998). Natural temperature regimes for loggerhead and green turtle nests in the eastern Mediterranean. *Canadian journal of zoology*, *76*(4), 723-729. https://doi.org/10.1139/z97-245
16. Casale, P., Gerosa, G., & Yerli, S. V. (2000). Female-biased primary sex ratio of the Green Turtle, C helonia mydas , estimated through sand temperatures at Akyatan, Turkey. *Zoology in the Middle East, 20*(1), 37–46. https://doi.org/10.1080/09397140.2000.10637810

17. Tomás, J., Godley, B. J., Castroviejo, J., & Raga, J. A. (2010). Bioko: critically important nesting habitat for sea turtles of West Africa. *Biodiversity and Conservation*, *19*, 2699-2714.
18. Trono, R. B. (1991). Philippine Marine Turtle Conservation Program. *Marine Turtle Newsletter, 53,* 5-7
19. Meylan, A. B., Brost, B., Conrad, L. J., Denison, S. H., Flaherty, D. B., Gray, J. A., Hardy, R. F., Meylan, P. A., Schwenter, J. A., Tornwall, B., & Owens, D. W. (2024). Feminization of a mixed-stock foraging aggregation of immature green turtles (Chelonia mydas), 1975–2018. *Marine Biology, 171*(1), 11. https://doi.org/10.1007/s00227-023-04320-2
20. Tolen, N., Rusli, M. U., & Booth, D. T. (2021). Relocating green turtle (Chelonia mydas) eggs to open beach areas produces highly female-biased hatchlings. *Herpetological Conservation and Biology*, *16*(3), 639-651.
21. Staines, M. N., Versace, H., Laloë, J., Smith, C. E., Madden Hof, C. A., Booth, D. T., Tibbetts, I. R., & Hays, G. C. (2023). Short-term resilience to climate-induced temperature increases for equatorial sea turtle populations. *Global Change Biology, 29*(23), 6546–6557. https://doi.org/10.1111/gcb.16952
22. Spotila, J. R., Standora, E. A., Morreale, S. J., & Ruiz, G. J. (1987). Temperature Dependent Sex Determination in the Green Turtle (*Chelonia mydas*): Effects on the Sex Ratio on a Natural Nesting Beach. *Herpetologica*, *43*(1), 74–81. http://www.jstor.org/stable/3892439
23. Standora, E. A., & Spotila, J. R. (1985). Temperature Dependent Sex Determination in Sea Turtles. *Copeia*, *1985*(3), 711–722. https://doi.org/10.2307/1444765
24. Esteban, N., Laloë, J.-O., Mortimer, J. A., Guzman, A. N., & Hays, G. C. (2016). Male hatchling production in sea turtles from one of the world's largest marine protected areas, the Chagos Archipelago. *Scientific Reports, 6*(1), 20339. https://doi.org/10.1038/srep20339
25. Calderón-Peña, R., Betancourt-Avila, R., Rodríguez-Fajardo, E., Martínez-González, Y., & Azanza Ricardo, J. (2020). Sex ratio of the green sea turtle Chelonia mydas (Testudines: Cheloniidae) hatchlings in the Guanahacabibes

Peninsula, Cuba. *Revista de Biología Tropical, 68*(3). https://doi.org/10.15517/rbt.v68i3.39033

26. Patrício, A., Marques, A., Barbosa, C., Broderick, A., Godley, B., Hawkes, L., Rebelo, R., Regalla, A., & Catry, P. (2017). Balanced primary sex ratios and resilience to climate change in a major sea turtle population. *Marine Ecology Progress Series, 577*, 189–203. https://doi.org/10.3354/meps12242
27. Tiwol, J. M. (2001). Sex ratios of hatchlings of the green turtle, *Chelonia mydas*, in natural nesting grounds, in open beach hatchery, and in sheltered beach hatchery in Gulisaan Island, Saba. *University Malaysia Sabah*.
28. Booth, D. T., & Astill, K. (2001). Temperature variation within and between nests of the green sea turtle, Chelonia mydas (Chelonia: Cheloniidae) on Heron Island, Great Barrier Reef. Australian Journal of Zoology, 49(1), 71. https://doi.org/10.1071/ZO00059
29. Booth, D. T., & Freeman, C. (2006). Sand and nest temperatures and an estimate of hatchling sex ratio from the Heron Island green turtle (Chelonia mydas) rookery, Southern Great Barrier Reef. Coral Reefs, 25(4), 629–633. https://doi.org/10.1007/s00338-006-0135-4
30. Miller, J. D., & Limpus, C. J. (1981). Incubation period and sexual differentiation in the green turtle *Chelonia mydas L*. In *Proceedings of the Melbourne Herpetological Symposium*, 66-73. The Zoological Board of Victoria: Melbourne
31. King, R., Cheng, W.-H., Tseng, C.-T., Chen, H., & Cheng, I.-J. (2013). Estimating the sex ratio of green sea turtles (Chelonia mydas) in Taiwan by the nest temperature and histological methods. *Journal of Experimental Marine Biology and Ecology, 445*, 140–147. https://doi.org/10.1016/j.jembe.2013.03.016
32. Godfrey, M. H., Mrosovsky, N., & Barreto, R. (1996). Estimating past and present sex ratios of sea turtles in Suriname. *Canadian Journal of Zoology, 74*(2), 267–277. https://doi.org/10.1139/z96-033
33. Bourjea, J., Dalleau, M., Derville, S., Beudard, F., Marmoex, C., Soili, A. M., ... & Frazier, J. (2015). Seasonality, abundance, and fifteen-year trend in green turtle nesting activity at Itsamia, Moheli, Comoros. *Endangered Species Research*, *27*(3), 265-276.

34. Jensen, M. P., Allen, C. D., Eguchi, T., Bell, I. P., LaCasella, E. L., Hilton, W. A., Hof, C. A. M., & Dutton, P. H. (2018). Environmental Warming and Feminization of One of the Largest Sea Turtle Populations in the World. *Current Biology, 28*(1), 154-159.e4. https://doi.org/10.1016/j.cub.2017.11.057
35. Swaminathan, A., Namboothri, N., & Shanker, K. (2011). Post-tsunami status of leatherback turtles on Little Andaman Island. *Indian Ocean Turtle Newsletter*, *14*, 5-10.
36. Stubbs, J. L., & Mitchell, N. J. (2018). The influence of temperature on embryonic respiration, growth, and sex determination in a Western Australian population of green turtles (*Chelonia mydas*). *Physiological and Biochemical Zoology*, *91*(6), 1102-1114. https://doi.org/10.1086/700433
37. Patrício, A. (2013). Habitat Selection and Climate Change Impacts on Green Turtles from Poilão Island, Guinea-Bissau. *University of Exeter*. https://ruffordorg.s3.amazonaws.com/media/project_reports/12317-1%20Detailed%20Final%20Report.pdf
38. Rebelo, R., Barbosa, C., Granadeiro, J. P., Indjai, B., Novais, B., Rosa, G. M., & Catry, P. (2012). Can leftovers from predators be reliably used to monitor marine turtle hatchling sex-ratios? The implications of prey selection by ghost crabs. *Marine Biology, 159*(3), 613–620. https://doi.org/10.1007/s00227-011-1839-8
39. Booth, D., Dunstan, A., Bell, I., Reina, R., & Tedeschi, J. (2020). Low male production at the world's largest green turtle rookery. Marine Ecology Progress Series, 653, 181–190. https://doi.org/10.3354/meps13500
40. Tanabe, L. K., Ellis, J., Elsadek, I., & Berumen, M. L. (2020). Potential feminization of Red Sea turtle hatchlings as indicated by in situ sand temperature profiles. *Conservation Science and Practice, 2*(10), e266. https://doi.org/10.1111/csp2.266
41. Stewart, T. A., Booth, D. T., & Rusli, M. U. (2019). Influence of sand grain size and nest microenvironment on incubation success, hatchling morphology and locomotion performance of green turtles (*Chelonia mydas*) at the Chagar Hutang Turtle Sanctuary, Redang Island, Malaysia. *Australian Journal of Zoology, 66*(6), 356. https://doi.org/10.1071/ZO19025

42. Laloë, J. O., Esteban, N., Berkel, J., & Hays, G. C. (2016). Sand temperatures for nesting sea turtles in the Caribbean: Implications for hatchling sex ratios in the face of climate change. *Journal of Experimental Marine Biology and Ecology*, *474*, 92-99.
43. Sönmez, B., Turan, C., Özdilek, Ş. Y., & Turan, F. (2016). Sex determination of green sea turtle (*Chelonia mydas*) hatchlings on the bases of morphological characters. *Journal of the Black Sea/Mediterranean Environment*
44. Özdilek, Ş. Y., Sönmez, B. E. K. T. A. Ş., & Kaska, Y. (2016). Sex ratio estimations of Chelonia mydas hatchlings at Samandağ Beach, Turkey. *Turkish Journal of Zoology, 40*(4), 552-560. http://dx.doi.org/10.3906/zoo-1501-17
45. Leh, C. M., Poon, S. K., & Siew, Y. C. (1985). Temperature-related phenomena affecting the sex of green turtle (Chelonia mydas) hatchlings in the Sarawak turtle islands. *Sarawak Museum Journal*, *34*(55), 183-193.
46. Burgess, E. A., Booth, D. T., & Lanyon, J. M. (2006). Swimming performance of hatchling green turtles is affected by incubation temperature. *Coral reefs*, *25*, 341-349. https://doi.org/10.1007/s00338-006-0116-7
47. Kılıç, Ç., & Candan, O. (2014). Hatchling sex ratio, body weight and nest parameters for Chelonia mydas nesting on Sugözü beaches (Turkey). *Animal Biodiversity and Conservation*, *37*(2), 177-182. https://doi.org/10.32800/abc.2014.37.0177
48. Candan, O., & Kolankaya, D. (2016). Sex Ratio of Green Turtle (Chelonia mydas) Hatchlings at Sugözü, Turkey: Higher Accuracy with Pivotal Incubation Duration. *Chelonian Conservation and Biology, 15*(1), 102–108. https://doi.org/10.2744/CCB-1132.1
49. Mrosovsky, N., Dutton, P. H., & Whitmore, C. P. (1984). Sex ratios of two species of sea turtle nesting in Suriname. *Canadian Journal of Zoology, 62*(11), 2227–2239. https://doi.org/10.1139/z84-324
50. Godfrey, M. H., & Mrosovsky, N. (2006). Pivotal temperature for green sea turtles, *Chelonia mydas*, nesting in Suriname. *The Herpetological Journal*, *16*(1), 55-61.
51. Laloë, J.-O., Monsinjon, J., Gaspar, C., Touron, M., Genet, Q., Stubbs, J., Girondot, M., & Hays, G. C. (2020). Production of male hatchlings at a remote South Pacific

green sea turtle rookery: Conservation implications in a female-dominated world. *Marine Biology, 167*(5), 70. https://doi.org/10.1007/s00227-020-03686-x

52. Velez-Espino, A., Pheasey, H., Araújo, A., & Fernández, L. M. (2018). Laying on the edge: demography of green sea turtles (Chelonia mydas) nesting on Playa Norte, Tortuguero, Costa Rica. *Marine Biology*, *165*, 1-12.
53. De Ocampo, G.D.D., Jaojoco, M., & Jaojoco, E.G. (1998). Incubation period, hatching percentage and sex ratio of green sea turtle (*Chelonia mydas L.*) hatchlings incubated at turtle islands, Philippines from April to June. *Philippine Journal of Veterinary Medicine*, *35*, 1 – 2. http://seaturtle.org/library/DeOcampoGD_1998_PhilJVetMed.pdf
54. Mrosovsky, N., Bass, A., Corliss, L. A., Richardson, J. I., & Richardson, T. H. (1992). Pivotal and beach temperatures for hawksbill turtles nesting in Antigua. *Canadian Journal of Zoology, 70*(10), 1920-1925. https://doi.org/10.1139/z92-261
55. Godfrey, M. H., D'Amato, A. F., Marcovaldi, M. Â., & Mrosovsky, N. (1999). Pivotal temperature and predicted sex ratios for hatchling hawksbill turtles from Brazil. *Canadian Journal of Zoology*, *77*(9), 1465-1473. https://doi.org/10.1139/z99-117
56. Dei Marcovaldi, M. A. G., Santos, A. J. B., Santos, A. S., Soares, L. S., Lopez, G. G., Godfrey, M. H., López-Mendilaharsu, M., & Fuentes, M. M. P. B. (2014). Spatio-temporal variation in the incubation duration and sex ratio of hawksbill hatchlings: Implication for future management. *Journal of Thermal Biology, 44*, 70–77. https://doi.org/10.1016/j.jtherbio.2014.06.010
57. Wibbels, T., Hillis-Starr, Z. M., & Phillips, B. (1999). Female-biased sex ratios of hatchling hawksbill sea turtles from a Caribbean nesting beach. *Journal of Herpetology*, *33*(1), 142-144. https://doi.org/10.2307/1565556
58. Burt, A. J., Dunn, N., Mason-Parker, C., Antha, S., & Mortimer, J. A. (2015). Curieuse National Park, Seychelles: critical management needs for protection of an important nesting habitat. *Marine Turtle Newsletter*, (147), 6.
59. Loop, K.A., Miller, J.D., & Limpus, C.J. (1995). Nesting by the hawsbill turtle (*Eretmochelys imbricata*) on Milman Island, Great Barrier Reef, Australia. *Wildlife Research, (22)*, 241-251. https://doi.org/10.1071/WR9950241

60. Dobbs, K.A., Miller, J.D., Limpus, C., & Landry, A.M. (2010). Hawksbill Turtle Hatchling Sex Ratios and Incubation and Pivotal Temperatures From Milman Island, Great Barrier Reef, Australia. *Marine Turtle Newsletter 128*, 12-16
61. Glen, F., & Mrosovsky, N. (2004). Antigua revisited: the impact of climate change on sand and nest temperatures at a hawksbill turtle (Eretmochelys imbricata) nesting beach. *Global Change Biology*, *10*(12), 2036-2045.
62. Simões, T. N., Silva, A. C. D., Santos, E. M. D., & Chagas, C. A. (2014). Temperatura de incubação e razão sexual em filhotes recém-eclodidos da tartaruga marinha Eretmochelys imbricata (Linnaeus, 1766) no município do Ipojuca, Pernambuco, Brasil. *Papéis Avulsos de Zoologia (São Paulo), 54*(25), 363–374. https://doi.org/10.1590/0031-1049.2014.54.25
63. Flores-Aguirre, C. D., Díaz-Hernández, V., Ugarte, I. H. S., & Caballero, L. E. S. (2020). Feminization tendency of Hawksbill Turtles (Eretmochelys imbricata) in the western Yucatán Peninsula, Mexico. *Amphib. Reptile Conserv., 14*(1).
64. Shaver, D. J., Owens, D. W., Chaney, A. H., Caillouet Jr, C. W., Burchfield, P., & Marquez, R. (1988). Styrofoam box and beach temperatures in relation to incubation and sex ratios of Kemp's ridley sea turtles. In *Schroeder BA (compiler):"The Eighth Annual Workshop on Sea Turtle Conservation and Biology." Fort Fisher, North Carolina: National Oceanic and Atmospheric Administration,* 103-108
65. Shaver, D. J., Rubio, C., Shelby Walker, J., George, J., Amos, A. F., Reich, K., ... & Shearer, T. (2016). Kemp's ridley sea turtle (Lepidochelys kempii) nesting on the Texas coast: Geographic, temporal, and demographic trends through 2014. *Gulf of Mexico Science*, *33*(2), 4.
66. Bevan, E. (2013). *Implications of hatchling sex ratios and survival in the recovery program for the endangered Kemp's ridley sea turtle* (Order No. 1543859). Available from ProQuest One Academic. (1433305582). http://ezproxy.deakin.edu.au/login?url=https://www.proquest.com/dissertations-theses/implications-hatchling-sex-ratios-survival/docview/1433305582/se-2
67. Hsu, S., & Shoemaker, A. (2018). Evaluating the Efficacy of Leatherback Turtle Conservation in Bocas Del Toro, Panama.

68. Patino-Martinez, J., Marco, A., Quiñones, L., & Hawkes, L. (2011). A potential tool to mitigate the impacts of climate change to the caribbean leatherback sea turtle. *Global Change Biology, 18*(2), 401–411. https://doi.org/10.1111/j.1385-2486.2011.02532.x
69. Binckley, C. A., Spotila, J. R., Wilson, K. S., & Paladino, F. V. (1998). Sex determination and sex ratios of Pacific leatherback turtles, *Dermochelys coriacea*. *Copeia*, 291-300. https://doi.org/10.2307/1447425
70. Steckenreuter, A., Pilcher, N., Krüger, B., & Ben, J. (2010). Male-Biased Primary Sex Ratio of Leatherback Turtles (Dermochelys coriacea) at the Huon Coast, Papua New Guinea. *Chelonian Conservation and Biology, 9*(1), 123–128. https://doi.org/10.2744/CCB-0763.1
71. Mast, R. B., Bailey, L. M. & Hutchinson, B. J. (Eds.). (2006). *SWOT report: The state of the world's sea turtles (Vol. 1)*. State of the World's Sea Turtles. https://www.seaturtlestatus.org/swot-report-1).
72. Revuelta, O., León, Y. M., Broderick, A. C., Feliz, P., Godley, B. J., Balbuena, J. A., Mason, A., Poulton, K., Savoré, S., Raga, J. A., & Tomás, J. (2015). Assessing the efficacy of direct conservation interventions: Clutch protection of the leatherback marine turtle in the Dominican Republic. *Oryx, 49*(4), 677–686. https://doi.org/10.1017/S0030605313001488
73. Rivas, M. L., Fernández, C. A. R. L. O. S., & Marco, A. (2016). Nesting ecology and population trend of leatherback turtles Dermochelys coriacea at Pacuare Nature Reserve, Costa Rica. *Oryx*, *50*(2), 274-282.
74. Sieg, A., Binckley, C., Wallace, B., Tomillo, P., Reina, R., Paladino, F., & Spotila, J. (2011). Sex ratios of leatherback turtles: Hatchery translocation decreases metabolic heating and female bias. *Endangered Species Research, 15*(3), 195–204. https://doi.org/10.3354/esr00372
75. Santidrián Tomillo, P., Oro, D., Paladino, F. V., Piedra, R., Sieg, A. E., & Spotila, J. R. (2014). High beach temperatures increased female-biased primary sex ratios but reduced output of female hatchlings in the leatherback turtle. *Biological Conservation, 176*, 71–79. https://doi.org/10.1016/j.biocon.2014.05.011
76. Neeman, N., Robinson, N. J., Paladino, F. V., Spotila, J. R., & O'Connor, M. P. (2015). Phenology shifts in leatherback turtles (Dermochelys coriacea) due to

changes in sea surface temperature. *Journal of Experimental Marine Biology and Ecology*, *462*, 113-120.

77. Metcalfe, K., Agamboué, P. D., Augowet, E., Boussamba, F., Cardiec, F., Fay, J. M., Formia, A., Kema, J. R. K., Kouerey, C., Mabert, B. D. K., Maxwell, S. M., Minton, G., Mounguengui, G.A.M., Moussounda, C., Moukoumou, N., Manfoumbi, J.C., Nguema, A.M., Nzegoue, N., Parnell, R.J., Plessis, P.D., Sounguet, G., Tilley, D., Verhage, S., Viljoen, W., White, L., Witt, M.J., & Godley, B. J. (2015). Going the extra mile: ground-based monitoring of olive ridley turtles reveals Gabon hosts the largest rookery in the Atlantic. *Biological Conservation*, *190*, 14-22.
78. Chan, E. H., & Liew, H. C. (1995). Incubation temperatures and sex-ratios in the Malaysian leatherback turtle Dermochelys coriacea. *Biological Conservation, 74*(3), 169–174. https://doi.org/10.1016/0006-3207(95)00027-2
79. Basford, S. J. (1988). *Temperature Regimes on Sandy Point, St. Croix and Implications for Sex Determination of Hatchlings Leatherback Sea Turtles* (Order No. 13852896). Available from ProQuest One Academic. (2203396608). http://ezproxy.deakin.edu.au/login?url=https://www.proquest.com/dissertations-theses/temperature-regimes-on-sandy-point-st-croix/docview/2203396608/se-2
80. Weston, E. G. (2013). Predicting leatherback sea turtle sex ratios using spatial interpolation of nesting beach temperatures. *The Charles E. Schmidt College of Science*
81. Rimblot, F., Fretey, J., Lescure, J., & Pieau, C. (1983, December). Influence de la temperature sur la differentiation sexuelle des gonades chez la tortue luth (Dermochelys coriacea); etude en incubation artificielle et naturelle. In *Bases biologiques de l'aquaculture, Montpellier,* 12-16
82. Rimblot, F., Fretey, J., Mrosovsky, N., Lescure, J., & Pieau, C. (1985). Sexual differentiation as a function of the incubation temperature of eggs in the sea-turtle *Dermochelys coriacea* (Vandelli, 1761). *Amphibia-Reptilia,* 6(1), 83-92. https://doi.org/10.1163/156853885X00218
83. Dutton, P. H., Whitmore, C. P., & Mrosovsky, N. (1985). Masculinisation of leatherback turtle Dermochelys coriacea hatchlings from eggs incubated in

styrofoam boxes. *Biological Conservation, 31*(3), 249–264. https://doi.org/10.1016/0006-3207(85)90070-9

84. Mast, R. B., Bailey, L. M., Hutchinson, B. J., & Hutchinson, A. (Eds.). (2007). *SWOT report: The state of the world's sea turtles* (Vol. 2). SWOT. https://www.seaturtlestatus.org/swot-report-vol-2
85. Godley, B. J., Broderick, A. C., & Mrosovsky, N. (2001). Estimating hatchling sex ratios of loggerhead turtles in Cyprus from incubation durations. *Marine Ecology Progress Series*, *210*, 195-201. http://dx.doi.org/10.3354/meps210195
86. Uçar, A. H., Kaska, Y., Ergene, S., Aymak, C., Kaçar, Y., Kaska, A., & İli, P. (2012). Sex Ratio Estimation of the Most Eastern Main Loggerhead Sea Turtle Nesting Site: Anamur Beach, Mersin, Turkey. *Israel Journal of Ecology & Evolution, 58*(1), 87–100. https://doi.org/10.1560/IJEE.58.1.87
87. Baldwin, R., Hughes, G. R., & Prince, R. I. T. (2003). Loggerhead turtles in the Indian Ocean. In, *Loggerhead sea turtles* (pp. 218–232). Smithsonian Books.
88. Georges, A., Limpus, C., & Stoutjesdijk, R. (1994). Hatchling sex in the marine turtle Caretta caretta is determined by proportion of development at a temperature, not daily duration of exposure. *Journal of Experimental Zoology*, *270*(5), 432-444. https://doi.org/10.1002/jez.1402700504
89. Dei Marcovaldi, M. Â., Godfrey, M. H., & Mrosovsky, N. (1997). Estimating sex ratios of loggerhead turtles in Brazil from pivotal incubation durations. *Canadian Journal of Zoology*, *75*(5), 755-770. https://doi.org/10.1139/z97-097
90. Dei Marcovaldi, M. A. G. dei, López-Mendilaharsu, M., Santos, A. S., Lopez, G. G., Godfrey, M. H., Tognin, F., Baptistotte, C., Thomé, J. C., Dias, A. C. C., De Castilhos, J. C., & Fuentes, M. M. P. B. (2016). Identification of loggerhead male producing beaches in the south Atlantic: Implications for conservation. *Journal of Experimental Marine Biology and Ecology, 477*, 14–22. https://doi.org/10.1016/j.jembe.2016.01.001
91. Hawkes, L. A., Broderick, A. C., Godfrey, M. H., & Godley, B. J. (2007). Investigating the potential impacts of climate change on a marine turtle population. *Global Change Biology, 0*(0), 070621084512044-??? https://doi.org/10.1111/j.1385-2486.2006.01320.x

92. Reneker, J. L., & Kamel, S. J. (2016). Climate change increases the production of female hatchlings at a northern sea turtle rookery. *Ecology, 97*(12), 3257-3264. https://doi.org/10.1002/ecy.1603

93. DeGregorio, B. A., & Williard, A. S. (2011). Incubation Temperatures and Metabolic Heating of Relocated and In Situ Loggerhead Sea Turtle (*Caretta caretta*) Nests at a Northern Rookery. *Chelonian Conservation and Biology, 10*(1), 54–61. https://doi.org/10.2744/CCB-0880.1

94. Mrosovsky, N., Hopkins-Murphy, S. R., & Richardson, J. I. (1984). Sex ratio of sea turtles: seasonal changes. *Science, 225*(4663), 739-741. https://doi.org/10.1126/science.225.4663.739

95. Wyneken, J., Epperly, S. P., Crowder, L. B., Vaughan, J., & Blair Esper, K. (2007). Determining sex in posthatchling loggerhead sea turtles using multiple gonadal and accessory duct characteristics. *Herpetologica*, *63*(1), 19-30. https://doi.org/10.1655/0018-0831(2007)63[19:DSIPLS]2.0.CO;2

96. Perez, E. A., Marco, A., Martins, S., & Hawkes, L. A. (2016). Is this what a climate change-resilient population of marine turtles looks like?. *Biological Conservation*, *193*, 124-132. http://dx.doi.org/10.1016/j.biocon.2015.11.023

97. Marco, A., Abella, E., Liria-Loza, A., Martins, S., López, O., Jiménez-Bordón, S., Medina, M., Oujo, C., Gaona, P., Godley, B. J., & López-Jurado, L. F. (2012). Abundance and exploitation of loggerhead turtles nesting in Boa Vista island, Cape Verde: the only substantial rookery in the eastern Atlantic. *Animal Conservation*, *15*(4), 351-360.

98. Lolavar, A., & Wyneken, J. (2015). Effect of rainfall on loggerhead turtle nest temperatures, sand temperatures and hatchling sex. *Endangered Species Research, 28*(3), 235–247. https://doi.org/10.3354/esr00684

99. Maxwell, J. A., Motara, M. A., & Frank, G. H. (1988). A micro-environmental study of the effect of temperature on the sex ratios of the loggerhead turtle, Caretta caretta , from Tongaland, Natal. *South African Journal of Zoology, 23*(4), 342–350. https://doi.org/10.1080/02541858.1988.11448123

100. Mrosovsky, N., & Provancha, J. (1989). Sex ratio of loggerhead sea turtles hatching on a Florida beach. *Canadian Journal of Zoology, 67*(10), 2533-2539. https://doi.org/10.1139/z89-358

101. Mrosovsky, N. A. J. P., & Provancha, J. (1992). Sex ratio of hatchling loggerhead sea turtles: data and estimates from a 5-year study. *Canadian Journal of Zoology*, *70*(3), 530-538. https://doi.org/10.1139/z92-080

102. Addison, D. S., & Morford, B. (1996). Sea turtle nesting activity on the Cay Sal Bank, Bahamas. *Bahamas Journal of Science*, *3*(3), 31-36.

103. Fuller, W., Godley, B., Hodgson, D., Reece, S., Witt, M., & Broderick, A. (2013). Importance of spatio-temporal data for predicting the effects of climate change on marine turtle sex ratios. *Marine Ecology Progress Series, 488*, 267–274. https://doi.org/10.3354/meps10419

104. Kaska, Y., Başkale, E., Katılmış, Y., & Urhan, R. (2005). Nest Temperatures and Sex Ratio Variations Among The Hatchlings And Embryos Of Loggerhead Turtles On Dalaman Beach, Turkey. *Proceedings, Second Mediterranean Conference on Marine Turtles, Kemer, 2005*

105. Başkale, E., & Kaska, Y. (2005). Sea turtle nest conservation techniques on southwestern beaches in Turkey. *Israel Journal of Ecology and Evolution*, *51*(1), 13-26.

106. Sarı, F., & Kaska, Y. (2016). Histochemical and immunohistochemical studies of the gonads and paramesonephric ducts of male and female hatchlings of loggerhead sea turtles (*Caretta caretta*). *Biotechnic & Histochemistry, 91*(6), 428–437. https://doi.org/10.1080/10520295.2016.1201143

107. Sarı, F., & Kaska, Y. (2015). Loggerhead sea turtle hatchling sex ratio differences between two nesting beaches in Turkey. *Israel Journal of Ecology and Evolution, 61*(3–4), 115–129. https://doi.org/10.1080/15659801.2015.1047681

108. Özdemir, A., Ilgaz, Ç., Durmuş, S. H., & Güçlü, Ö. (2011). The effect of the predicted air temperature change on incubation temperature, incubation duration, sex ratio and hatching success of loggerhead turtles. *Animal Biology*, *61*(4), 369-383. http://doi.org/10.1163/157075511x596864

109. Ilgaz, Ç. E. T. İ. N., Özdemir, A., Kumlutaş, Y., & Durmuş, S. H. (2011). The effect of nest relocation on embryonic mortality and sex ratio of Loggerhead Turtles, Caretta caretta (Reptilia: Cheloniidae), at Dalyan Beach, Turkey. Italian

Journal of Zoology, 78(3), 354–363.
https://doi.org/10.1080/11250003.2010.509742

110. Reinhold, L., & Whiting, A. (2014). High-density loggerhead sea turtle nesting on Dirk Hartog Island, Western Australia. *Marine Turtle Newsletter*, (141), 7.

111. Kaska, Y., Ilgaz, Ç., Özdemir, A., Başkale, E., Türkozan, O., Baran, İ., & Stachowitsch, M. (2006). Sex ratio estimations of loggerhead sea turtle hatchlings by histological examination and nest temperatures at Fethiye beach, Turkey. *Naturwissenschaften, 93*(7), 338–343. https://doi.org/10.1007/s00114-006-0110-5

112. Wyneken, J., & Lolavar, A. (2015). Loggerhead sea turtle environmental sex determination: Implications of moisture and temperature for climate change based predictions for species survival. *Journal of Experimental Zoology Part B: Molecular and Developmental Evolution, 324*(3), 295–314.
https://doi.org/10.1002/jez.b.22620

113. Mrosovsky, N. (1988). Pivotal temperatures for loggerhead turtles (*Caretta caretta*) from northern and southern nesting beaches. *Canadian Journal of Zoology*, *66*(3), 661-669. https://doi.org/10.1139/z88-098

114. Richards, P. M., Epperly, S. P., Heppell, S. S., King, R. T., Sasso, C. R., Moncada, F., Nodarse, G., Shaver, D. J., Medina, Y., & Zurita, J. (2011). Sea turtle population estimates incorporating uncertainty: a new approach applied to western North Atlantic loggerheads Caretta caretta. *Endangered Species Research*, *15*(2), 151-158.

115. Dodd, C. K. (1988). *Synopsis of the biological data on the loggerhead sea turtle: Caretta caretta (Linnaeus, 1758)* (Vol. 88, No. 14). Fish and Wildlife Service, US Department of the Interior.

116. Thomson, J. A., Hajnoczky, N., & Hattingh, K. (2016). The sea turtle rookery at Gnaraloo Bay, Western Australia: using nocturnal observations to validate diurnal track interpretations. *Chelonian Conservation and Biology*, *15*(2), 187-196.

117. Sari, F., & Kaska, Y. (2017). Assessment of hatchery management for the loggerhead turtle (Caretta caretta) nests on Göksu Delta, Turkey. *Ocean &*

Coastal Management, 146, 89–98. https://doi.org/10.1016/j.ocecoaman.2017.06.010

118. Limpus, C. J., Reed, P. C., & Miller, J. D. (1985). Temperature dependent sex determination in Queensland sea turtles: intraspecific variation in Caretta caretta. *Biology of Australasian frogs and reptiles*, *1985*, 343-351.

119. Hanson, J., Wibbels, T., & Martin, R. E. (1998). Predicted female bias in sex ratios of hatchling loggerhead sea turtles from a Florida nesting beach. *Canadian Journal of Zoology, 76*(10), 1850-1861. https://doi.org/10.1139/z98-118

120. Matsuzawa, Y., Sato, K., Tanaka, H., Bando, T., Sakamoto, W., & Gotou, K. (1998). Estimation of sex ratio of loggerhead turtles hatching on the Senri-coast in Japan. In *Proceedings of the Sixteenth Annual Symposium on Sea Turtle Biology and Conservation. NOAA Tech. Rep. NMFS-SEFSC-412* (pp. 101-102).

121. Schmid, J. L., Addison, D. S., Donnelly, M. A., Shirley, M. A., & Wibbels, T. (2008). The Effect of Australian Pine (Casuarina equisetifolia) Removal on Loggerhead Sea Turtle (Caretta caretta) Incubation Temperatures on Keewaydin Island, Florida. *Journal of Coastal Research, 10055*, 214–220. https://doi.org/10.2112/SI55-001.1

122. Houghton, J. D. R., & Hays, G. C. (2001). Asynchronous emergence by loggerhead turtle (Caretta caretta) hatchlings. *Naturwissenschaften*, *88*, 133-136. https://doi.org/10.1007/s001140100212

123. Kobayashi, S., Wada, M., Fujimoto, R., Kumazawa, Y., Arai, K., Watanabe, G., & Saito, T. (2017). The effects of nest incubation temperature on embryos and hatchlings of the loggerhead sea turtle: Implications of sex difference for survival rates during early life stages. *Journal of Experimental Marine Biology and Ecology, 486*, 274–281. https://doi.org/10.1016/j.jembe.2016.10.020

124. Jribi, I., & Bradai, M. N. (2014). Sex ratio estimations of loggerhead sea turtle hatchlings at Kuriat Islands, Tunisia: Can minor nesting sites contribute to compensate globally female-biased sex ratio?. *The Scientific World Journal*, *2014*(1), 419410. https://doi.org/10.1155/2014/419410

125. Rees, A. F., & Margaritoulis, D. (2004). Beach temperatures, incubation durations and estimated hatchling sex ratio for loggerhead sea turtle nests in southern Kyparissia Bay, Greece. *Testudo*, 6(1), 23-36.

126. Mrosovsky, N., Kamel, S., Rees, A. F., & Margaritoulis, D. (2002). Pivotal temperature for loggerhead turtles (*Caretta caretta*) from Kyparissia Bay, Greece. *Canadian Journal of Zoology*, *80*(12), 2118-2124. https://doi.org/10.1139/z02-204

127. Read, T., Booth, D. T., & Limpus, C. J. (2013). Effect of nest temperature on hatchling phenotype of loggerhead turtles (Caretta caretta) from two South Pacific rookeries, Mon Repos and La Roche Percée. *Australian Journal of Zoology*, *60*(6), 402-411. https://doi.org/10.1071/ZO12079

128. Wood, A., Booth, D. T., & Limpus, C. J. (2014). Sun exposure, nest temperature and loggerhead turtle hatchlings: Implications for beach shading management strategies at sea turtle rookeries. *Journal of Experimental Marine Biology and Ecology, 451*, 105–114. https://doi.org/10.1016/j.jembe.2013.11.005

129. Chu, C.T., Booth, D.T., & Limpus, C.J. (2008). Estimating the sex ratio of loggerhead turtle hatchlings at Mon Repos rookery (Australia) from nest temperatures. *Australian Journal of Zoology*, *56*(1), 57-64. https://doi.org/10.1071/ZO08004

130. Gross, T. S., Crain, D. A., Bjorndal, K. A., Bolten, A. B., & Carthy, R. R. (1995). Identification of sex in hatchling loggerhead turtles (Caretta caretta) by analysis of steroid concentrations in chorioallantoic/amniotic fluid. *General and comparative endocrinology*, *99*(2), 204-210. https://doi.org/10.1006/gcen.1995.1103

131. Öz, M., Erdoğan, A., Kaska, Y., Düşen, S., Aslan, A. Z. İ. Z., Sert, H. A. K. A. N., Yavuz, M., & Tunc, M. R. (2004). Nest temperatures and sex-ratio estimates of loggerhead turtles at Patara beach on the southwestern coast of Turkey. *Canadian Journal of Zoology*, *82*(1), 94-101. http://dx.doi.org/10.1139/z03-200

132. Fuentes, M. M. P. B., Monsinjon, J., Lopez, M., Lara, P., Santos, A., Dei Marcovaldi, M. A. G., & Girondot, M. (2017). Sex ratio estimates for species with temperature-dependent sex determination differ according to the proxy used. *Ecological Modelling, 365*, 55–67. https://doi.org/10.1016/j.ecolmodel.2017.09.022

133. Limpus, C. J. (1985). A study of the loggerhead turtle, *Caretta caretta*, in Queensland (Doctoral thesis, University of Queensland). University of Queensland.

134. Taix-Bonnin, R. E., Farman, R., Géraux, H., & Faninoz, S. (2011). Conservation et suivi des populations de tortues marines en Nouvelle-Calédonie. *Bulletin de la Société herpétologique de France*, (139-40), 151-165.

135. Laloë, J.-O., Cozens, J., Renom, B., Taxonera, A., & Hays, G. C. (2014). Effects of rising temperature on the viability of an important sea turtle rookery. *Nature Climate Change, 4*(6), 513–518. https://doi.org/10.1038/nclimate2236

136. Laloë, J. O., Cozens, J., Renom, B., Taxonera, A., & Hays, G. C. (2017). Climate change and temperature-linked hatchling mortality at a globally important sea turtle nesting site. *Global change biology*, *23*(11), 4922-4931.

137. Rocha, P. R., Melo, T., Rebelo, R., & Catry, P. (2015). A Significant Nesting Population of Loggerhead Turtles at the Nature Reserve of Santa Luzia, Cabo Verde. *Chelonian Conservation and Biology, 14*(2), 161–166. https://doi.org/10.2744/CCB-1143.1

138. Jribi, I., Hamza, A., Saied, A., & Ouergui, A. (2013). Sex ratio estimations of loggerhead marine turtle hatchlings by incubation duration and nest temperature at Sirte beaches (Libya). *Scientia Marina, 77*(4), 617–624. https://doi.org/10.3989/scimar.03855.28B

139. Tanner, C., Marco, A., Martins, S., Abella-Perez, E., & Hawkes, L. (2019). Highly feminised sex-ratio estimations for the world's third-largest nesting aggregation of loggerhead sea turtles. *Marine Ecology Progress Series, 621*, 209–219. https://doi.org/10.3354/meps12963

140. Yntema, C. L., & Mrosovsky, N. (1982). Critical periods and pivotal temperatures for sexual differentiation in loggerhead sea turtles. *Canadian Journal of Zoology*, *60*(5), 1012-1016. https://doi.org/10.1139/z82-141

141. LeBlanc, A. M., Drake, K. K., Williams, K. L., Frick, M. G., Wibbels, T., & Rostal, D. C. (2012). Nest Temperatures and Hatchling Sex Ratios from Loggerhead Turtle Nests Incubated Under Natural Field Conditions in Georgia, United States. *Chelonian Conservation and Biology, 11*(1), 108–116. https://doi.org/10.2744/CCB-0915.1

142. Mrosovsky, N. (1982). Sex ratio bias in hatchling sea turtles from artificially incubated eggs. *Biological Conservation, 23*(4), 309–314. https://doi.org/10.1016/0006-3207(82)90087-8

143. Katselidis, K. A., Schofield, G., Stamou, G., Dimopoulos, P., & Pantis, J. D. (2012). Females first? Past, present and future variability in offspring sex ratio at a temperate sea turtle breeding area. *Animal Conservation, 15(*5), 508–518. https://doi.org/10.1111/j.1469-1795.2012.00543.x

144. Zbinden, J., Davey, C., Margaritoulis, D., & Arlettaz, R. (2007). Large spatial variation and female bias in the estimated sex ratio of loggerhead sea turtle hatchlings of a Mediterranean rookery. *Endangered Species Research, 3*, 305–312. https://doi.org/10.3354/esr00058

145. Maulany, R. I., Booth, D. T., & Baxter, G. S. (2012). Emergence Success and Sex Ratio of Natural and Relocated Nests of Olive Ridley Turtles from Alas Purwo National Park, East Java, Indonesia. *Copeia, 2012*(4), 738–747. https://doi.org/10.1643/CH-12-088

146. Castheloge, V.D., dos Santos, M.R.D.D., de Castilhos, J.C., Paulo Roberto de J. Filho, P.R.D.J., Gomes, L.D.C., Clemente-Carvalho, R.B.G., & Ferreira, P.D. (2018). Pivotal Temperature and Hatchling Sex Ratio of Olive Ridley Sea Turtles *Lepidochelys olivacea* from the South Atlantic Coast of Brazil. *Herpetological Conservation and Biology 13*(2):488–496.

147. McCoy, C. J., Vogt, R. C., & Censky, E. J. (1983). Temperature-controlled sex determination in the sea turtle Lepidochelys olivacea. *Journal of Herpetology*, *17*(4), 404-406. https://doi.org/10.2307/1563594

148. Wibbels, T., Rostal, D., & Byles, R. (1998). High Pivotal Temperature in the Sex Determination of the Olive Ridley Sea Turtle, *Lepidochelys olivacea*, from Playa Nancite, Costa Rica. *Copeia*, *1998*(4), 1086–1088. https://doi.org/10.2307/1447364

149. Wen, F. (2018). Field Pivotal Temperature and Hatchling Sex Ratios of Olive Ridley Sea Turtles (*Lepidochelys olivacea*) at Ostional Beach, Costa Rica (Order No. 10843448). Available from ProQuest One Academic. (2088909558). http://ezproxy.deakin.edu.au/login?url=https://www.proquest.com/dissertations

-theses/field-pivotal-temperature-hatchling-sex-ratios/docview/2088909558/se-2

150. Das, D., & Mandal, S. (2019). Unusual death of an Olive Ridley Sea Turtle in Dhabaleswar sea beach, Odisha, India. *Journal of Bombay Natural History Society*, *116*, 7-10.

151. Mohanty-Hejmadi, P., Behra, M., & Dimond, M. T. (1985). Temperature dependent sex differentiation in the olive ridley *Lepidochelys olivacea* and its implications for conservation. In *Symposium on endangered marine animals and marine parks. Cochin. Marine Biological Association of India*,1-5

152. Hernández-Echeagaray, O. E., Hernández-Cornejo, R., Harfush-Meléndez, M., & García-Gasca, A. (2012). Evaluation of sex ratios of the olive ridley sea turtle (Lepidochelys olivacea) on the arribada nesting beach, La Escobilla, Mexico. *Marine Turtle Newsletter*, *133*, 12-16.

153. González, C. V., Bátiz, F. S., & Vázquez, S. H. (2012). Proporción sexual en crías de la tortuga marina Lepidochelys olivacea, producida en corral de incubación en la playa de anidación La Gloria, Jalisco, México. *Boletín del Centro de Investigaciones Biológicas*, *34*(3).

154. Tello-Sahagún, L. A., Ley-Quiñonez, C. P., Abreu-Grobois, F. A., Monsinjon, J. R., Zavala-Norzagaray, A. A., Girondot, M., & Hart, C. E. (2023). Neglecting cooler low-season nest protection could deprive sea turtle populations of valuable hatchlings. *Biological Conservation*, *277*, 109873. https://doi.org/10.1016/j.biocon.2022.109873

155. Binhammer, M.R., Beange, M., Arauz, R. (2019). Sand Temperature, Sex Ratios, and Nest Success in Olive Ridley Sea Turtles. *Marine Turtle Newsletter*, 159:5-9, http://www.seaturtle.org/mtn/archives/mtn159/mtn159-2.shtml

156. Garcıía, A., Ceballos, G., & Adaya, R. (2003). Intensive beach management as an improved sea turtle conservation strategy in Mexico. *Biological Conservation, 111*(2), 253–261. https://doi.org/10.1016/S0006-3207(02)00300-2

157. Robledo-Avila, L. A., Phillips-Farfán, B. V., Harfush Meléndez, M., Lopez Toledo, L., Tafolla Venegas, D., Herrera Vargas, Ma. A., Ruíz Cortés, D. V., & Meléndez-Herrera, E. (2022). Short communication: Ex-situ conservation in

hatcheries is associated with spleen development in Lepidochelys olivacea turtle hatchlings. *Comparative Biochemistry and Physiology Part A: Molecular & Integrative Physiology, 265*, 111130. https://doi.org/10.1016/j.cbpa.2021.111130
